# Breast Cancer: Epidemiology, Molecular Classification, Diagnostics and Evolving Treatment Paradigms

**DOI:** 10.3390/molecules31142551

**Published:** 2026-07-22

**Authors:** Jeremiah Oshiomame Unuofin, Adedoyin Omobolanle Adefisan-Adeoye, Oluwatomiwa Kehinde Paimo, Nhlanhla Maphetu, Sogolo Lucky Lebelo

**Affiliations:** 1 Department of Life and Consumer Sciences, University of South Africa, Johannesburg 1709, South Africa; bonganimapheto@gmail.com (N.M.); lebelol@unisa.ac.za (S.L.L.); 2 Chemical Sciences Department, Faculty of Computing and Applied Sciences, Dominion University, Ibadan 200243, Nigeria; adedoyinadefisan22@gmail.com; 3 Department of Pediatric Brain Tumors Research Program, Hematology-Oncology Section, Baylor College of Medicine, Houston, TX 77030, USA; 4 Department of Biochemistry, College of Biosciences, Federal University of Agriculture, Abeokuta 110111, Nigeria; ktomiwa@gmail.com

**Keywords:** breast cancer, breast cancer diagnosis, cancer genetics, gene therapy, traditional medicine, next-generation sequencing, triple-negative breast cancer

## Abstract

Breast cancer remains one of the most prevalent malignancies affecting women worldwide and continues to be a leading cause of cancer-related morbidity and mortality. Patients may present with either localized or advanced disease, with clinical outcomes increasingly influenced by molecular subtype and genetic profile. This review highlights the key genetic factors involved in breast cancer, current diagnostic and therapeutic strategies, and promising emerging approaches that may shape future clinical management. Breast cancer diagnosis typically involves clinical breast examination, imaging techniques such as mammography and ultrasound, and confirmatory biopsies. Genetic mutations in specific genes are strongly linked to the development, progression, and metastasis of the disease. Treatment options for localized breast cancer continue to include surgery (lumpectomy or mastectomy) and radiotherapy, combined with systemic therapies tailored to tumor biology, such as endocrine therapy, human epidermal growth factor receptor 2 (HER2)-targeted therapy, and cyclin-dependent kinase (CDK)4/6 inhibitors. For advanced or metastatic breast cancer, recent therapeutic advances include the use of immunotherapy (e.g., immune checkpoint inhibitors), Poly (ADP-ribose) polymerase (PARP) inhibitors for Breast Cancer gene (BRCA)-mutated cancers, antibody–drug conjugates, and novel targeted agents, which have significantly improved patient outcomes in selected populations. Recent findings in breast cancer genetics have highlighted the critical role of germline and somatic mutations, particularly in genes such as BRCA1, BRCA2, phosphatidylinositol-4,5-bisphosphate 3-kinase catalytic subunit alpha (PIK3CA), and TP53, in driving tumor initiation, progression, and therapeutic response. Molecular profiling and next-generation sequencing technologies have enabled more precise tumor classification and facilitated the development of personalized treatment strategies. Despite these advances, treatment resistance and disease recurrence remain major challenges, particularly in aggressive subtypes such as triple-negative breast cancer. Consequently, ongoing research is exploring alternative and complementary approaches, including nanotechnology-based drug delivery systems, gene editing techniques such as clustered regularly interspaced short palindromic repeats-Cas9 (CRISPR-associated protein 9) (CRISPR-Cas9), cancer vaccines, and the integration of traditional and plant-derived compounds. These strategies aim to enhance therapeutic efficacy, reduce systemic toxicity, and overcome resistance mechanisms.

## 1. Introduction

Breast cancer (BC) remains the most commonly diagnosed malignancy among women worldwide and a leading cause of cancer-related morbidity and mortality [1,2]. According to the latest global estimates, breast cancer accounted for approximately 2.3 million new cases worldwide in 2022, representing around 11.6% of all newly diagnosed cancers, and was responsible for approximately 670,000 deaths [2]. Despite considerable advances in diagnosis and treatment, the global burden of breast cancer continues to increase. Recent projections indicate that the number of new breast cancer cases and related deaths will rise substantially by 2050, with the greatest increases expected in low- and middle-income countries (LMICs) because of population growth, ageing, urbanisation, and changing reproductive and lifestyle patterns [3,4]. In Africa alone, an estimated 198,000 new breast cancer cases and more than 91,000 deaths were reported in 2022, highlighting persistent disparities in cancer outcomes across the continent [3].

Breast cancer incidence and survival are influenced by a complex interaction of genetic susceptibility, reproductive and hormonal factors, environmental exposures, socioeconomic conditions, lifestyle behaviours, and access to healthcare services [5,6]. In high-income countries, the increasing incidence of breast cancer has been associated with delayed childbearing, lower parity, reduced breastfeeding, prolonged exposure to endogenous and exogenous hormones, obesity, alcohol consumption, and physical inactivity [6]. In contrast, the rising burden of breast cancer in LMICs is primarily driven by demographic transition, urbanisation, changing reproductive behaviours, limited awareness, and inadequate access to screening and treatment rather than inherited genetic mutations alone [3,6]. Consequently, delayed diagnosis, poor screening coverage, and restricted access to effective treatment contribute substantially to the higher mortality observed in these settings [3].

Marked disparities in breast cancer outcomes are also observed across racial and ethnic populations. Although the overall incidence of breast cancer is generally lower among Black women than White women in many high-income countries, Black women experience approximately 40–42% higher breast cancer mortality owing to a combination of socioeconomic inequities, barriers to healthcare access, delayed diagnosis, reduced treatment opportunities, and a higher prevalence of aggressive tumor subtypes, particularly triple-negative breast cancer [7,8,9]. Additional prognostic factors, including age at diagnosis, body mass index, tumor biology, comorbidities, and treatment adherence, further contribute to variations in clinical outcomes [10].

Based on histopathological features, breast cancer is broadly classified as either pre-invasive or invasive [1,11]. The principal pre-invasive lesions include ductal carcinoma *in situ* (DCIS) and lobular carcinoma *in situ* (LCIS), whereas invasive breast cancers predominantly comprise invasive ductal carcinoma (IDC) and invasive lobular carcinoma (ILC), which together account for the vast majority of breast cancer diagnoses. These histological subtypes exhibit distinct pathological, molecular, and clinical characteristics that influence prognosis and therapeutic decision-making [1].

Breast cancer management has evolved considerably over the past decade and currently encompasses surgery (breast-conserving surgery or mastectomy), radiotherapy, chemotherapy, endocrine therapy, targeted therapies, immunotherapy, antibody–drug conjugates (ADCs), and other precision medicine approaches selected according to tumor stage, molecular subtype, and patient-specific characteristics [12,13,14]. Although these therapeutic advances have significantly improved patient survival and quality of life, many treatments remain associated with substantial toxicity, acquired resistance, and variable clinical responses.

Despite remarkable progress in breast cancer diagnosis and treatment, important knowledge gaps remain regarding the complex interplay between molecular genetics, tumor heterogeneity, the tumor microenvironment, and clinical characteristics that collectively influence disease initiation, progression, therapeutic response, and patient outcomes. Integrating these interconnected biological and clinical factors is essential for improving risk stratification, early diagnosis, personalised treatment selection, and long-term disease management.

This review provides a comprehensive overview of the current understanding of breast cancer pathology, molecular and genetic mechanisms, diagnostic approaches, and established as well as emerging therapeutic strategies. In addition, it discusses the challenges and future directions of precision oncology, highlighting recent advances in molecular classification, biomarker development, and novel targeted therapies aimed at improving patient outcomes.

## 2. Epidemiology

### 2.1. Global Scale

Breast cancer remains the most commonly diagnosed malignancy and the leading cause of cancer-related mortality among women worldwide. According to GLOBOCAN 2022 estimates, there were approximately 2.3 million new cases and 670,000 deaths, accounting for 11.5% of all new cancer diagnoses and 6.8% of cancer-related deaths globally [2,15].

The global burden of breast cancer is characterised by marked geographic disparities in both incidence and outcomes. Although high-income countries report higher incidence rates, they have experienced sustained reductions in mortality due to organised screening programmes, early detection, and improved access to multimodal treatment strategies. In contrast, low- and middle-income countries (LMICs) continue to experience increasing incidence without a corresponding decline in mortality. This disparity is largely driven by demographic transition, urbanisation, changes in reproductive patterns, and increasing prevalence of modifiable lifestyle risk factors, alongside limited access to early detection and effective treatment services [2,4].

These inequalities are further reflected in mortality-to-incidence ratios (MIRs), which serve as a proxy indicator of cancer survival derived from GLOBOCAN age-standardised incidence and mortality estimates. Regions with limited healthcare infrastructure consistently demonstrate higher MIRs, reflecting delayed diagnosis, inadequate pathology and imaging services, and restricted access to cancer treatment. In contrast, lower MIRs observed in high-income regions highlight the effectiveness of established cancer control programmes, including screening and integrated treatment pathways [2,16].

Collectively, these patterns underscore persistent global inequities in breast cancer outcomes despite advances in diagnostic and therapeutic strategies, emphasising the urgent need for strengthened cancer control systems, particularly in resource-limited settings.

### 2.2. Local Scale

In Africa, breast cancer disparities are particularly pronounced, with incidence rates continuing to rise steadily across many regions [17]. Although incidence remains lower than in high-income Western countries, mortality-to-incidence ratios (MIRs) are among the highest globally, reflecting substantial inequities in cancer outcomes [2,15]. This pattern is largely driven by late-stage presentation, with approximately 60–70% of patients diagnosed at stage III or IV, primarily due to limited access to screening services, delayed diagnosis, financial barriers to care, and sociocultural factors influencing health-seeking behaviour [4,16].

In addition to late presentation, tumor biology contributes to poor outcomes in the region. Studies indicate a relatively higher proportion of aggressive subtypes, particularly triple-negative breast cancer (TNBC), although variability in diagnostic capacity and pathology infrastructure may influence subtype reporting consistency [5,8]. Combined with delayed diagnosis, these factors contribute to the disproportionately high breast cancer mortality observed across African populations [18].

Nigeria reflects the broader breast cancer burden observed across sub-Saharan Africa. Recent registry-based analyses indicate a rising incidence, attributed to rapid urbanisation, reproductive pattern shifts, increasing obesity prevalence, and adoption of westernised lifestyles [19,20]. However, survival outcomes remain poor due to fragmented referral pathways, limited oncology infrastructure, inadequate pathology services, and the absence of nationwide screening programmes [16,21]. In addition, incomplete cancer registry coverage contributes to underreporting of incidence and mortality, thereby limiting accurate national cancer burden estimation and planning [15].

Country-specific breast cancer incidence and mortality estimates (per 100,000 population) were obtained from the GLOBOCAN 2022 database, developed by the International Agency for Research on Cancer (IARC). Age-standardised incidence rates (ASIR) and age-standardised mortality rates (ASMR) were extracted for all countries with available data.

For each country, the mortality-to-incidence ratio (MIR) was calculated using the formula:

MIR = ASMR/ASIR

where ASMR represents the age-standardised mortality rate, and ASIR represents the age-standardised incidence rate.

The MIR is a population-level indicator reflecting the relationship between cancer mortality and incidence and is commonly used as a proxy measure of cancer outcomes and healthcare system performance at the population level. To minimize the influence of differences in population age structures across countries, MIR values were derived using age-standardised rather than crude rates.

Countries were subsequently ranked according to MIR values, and global patterns were visualized using choropleth maps generated in R (version 4.5.1). Descriptive analyses were performed to summarize regional variation in MIR values and to identify countries with comparatively high and low mortality burdens relative to incidence.

**Figure 1:**
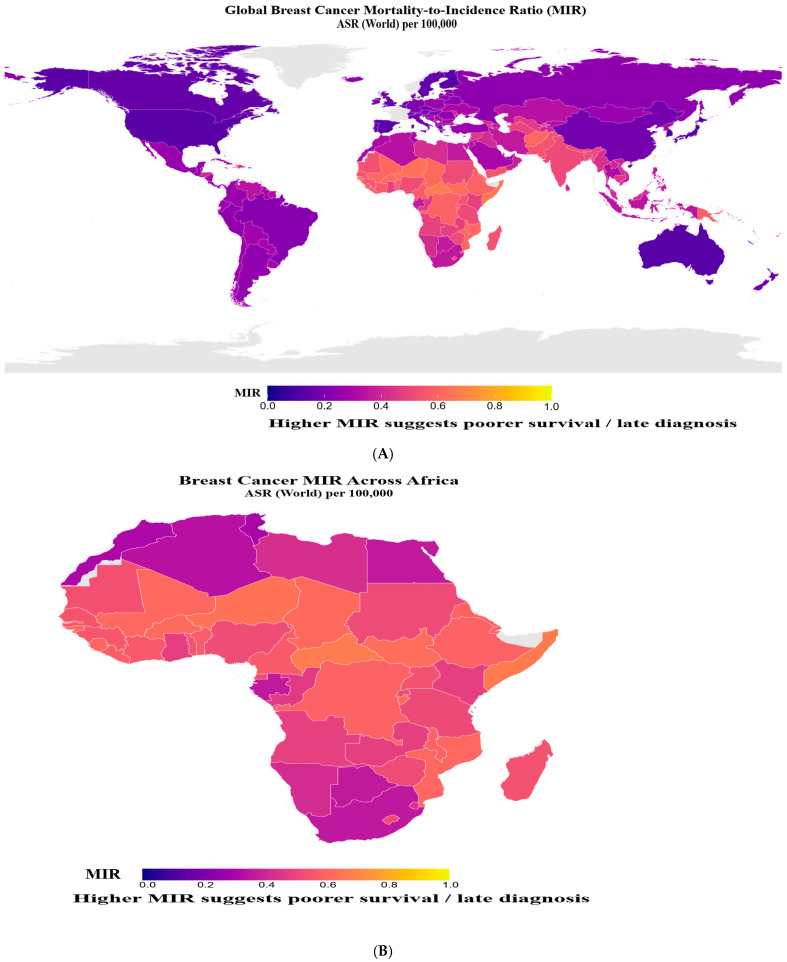
Original choropleth maps illustrating breast cancer mortality-to-incidence ratios (MIRs) at the (**A**) global level and (**B**) in Africa. Higher MIR values are associated with late/delayed diagnosis, limited access to population-based screening, inadequate pathology and imaging services, and restricted availability of multimodal treatment, particularly in low- and middle-income countries, and serve as a proxy for poorer survival outcomes. In contrast, lower MIRs observed in high-income regions reflect the impact of organised screening programmes and broader access to comprehensive cancer care. Data were synthesised from GLOBOCAN 2022 estimates.

Consistent with these observations, elevated mortality-to-incidence ratios across African countries, as illustrated in Figure 1, further reinforce the association between weak health systems and poor breast cancer outcomes. Collectively, these findings highlight the urgent need for context-specific interventions aimed at strengthening early detection, expanding diagnostic capacity, improving access to multidisciplinary care, and enhancing cancer registry infrastructure across the region.

## 3. Quantitative Summary of Global and Regional Breast Cancer MIR Patterns

The global distribution of the breast cancer mortality-to-incidence ratio (MIR), derived from GLOBOCAN 2022 age-standardised incidence and mortality rates, is illustrated in Figure 1 and Table 1 and demonstrates substantial regional variation in outcomes. MIR values vary widely across countries, reflecting differences in healthcare access, early detection capacity, and treatment availability [2,15].

High-income regions, including North America, Western Europe, and Oceania, generally exhibit lower MIR values (≤0.20), indicating more favourable survival patterns due to organised screening programmes, early-stage diagnosis, and access to multimodal treatment. Countries such as the United States (0.127), Norway (0.115), Australia (0.121), and Belgium (0.136) fall within this lower MIR range, consistent with well-developed cancer control systems [15,16]. These areas make up a sizable percentage of nations with MIR < 0.20 and are seen on the heat map as darker purple tones, as shown in Figure 1.

In contrast, higher MIR values (≥0.50) are more frequently observed in low- and middle-income countries, particularly across sub-Saharan Africa and parts of South and Southeast Asia. These elevated ratios are indicative of delayed diagnosis, limited access to diagnostic pathology and imaging services, and constrained treatment capacity rather than direct fatality proportions. Countries such as the Central African Republic (0.671), Somalia (0.666), Niger (0.644), and Fiji (0.645) are consistently reported among the highest MIR settings globally, reflecting significant structural limitations in cancer care delivery [2,4]. Significantly, Table 1 shows that almost 60% of African nations had MIR values higher than 0.50, indicating a disproportionate burden on the continent.

Africa demonstrates the highest regional MIR burden globally. Many sub-Saharan African countries exhibit MIR values between approximately 0.50 and 0.65, highlighting persistent inequalities in cancer outcomes. Nigeria (0.520), Ethiopia (0.588), Côte d’Ivoire (0.559), and Senegal (0.548) also demonstrate elevated MIRs, consistent with late-stage presentation and limited access to early detection services. Although incidence rates are lower than in high-income regions, mortality remains disproportionately high, underscoring the impact of health system constraints on survival outcomes [2,16].

A smaller proportion of countries in Eastern Europe, Latin America, and parts of Asia fall within an intermediate MIR range (0.20–0.40), including Brazil, China, and Turkey, reflecting heterogeneous access to cancer care and screening infrastructure. These variations further emphasise the gradient between health system capacity and breast cancer outcomes.

Overall, the heat map’s distinct gradient from low MIR locations (purple) to high MIR regions (yellow) visually supports the quantitative patterns shown in Table 1. The observed global gradient from low MIR regions (high-income countries) to high MIR regions (resource-limited settings) underscores a strong inverse association between MIR and healthcare system strength. This pattern highlights persistent global inequities in breast cancer outcomes and reinforces the urgent need for improved early detection, diagnostic infrastructure, and equitable access to effective treatment, particularly in high-MIR regions such as sub-Saharan Africa.

## 4. Types of Breast Cancer

Breast cancer comprises a heterogeneous group of malignancies arising from different anatomical compartments of the breast, including the ducts, lobules, and surrounding stromal tissue. Contemporary histopathological classification recognises breast cancer as a highly diverse disease with substantial inter- and intra-tumor heterogeneity influencing diagnosis, prognosis, and treatment response [22,23].

From a broad pathological perspective, breast cancers are classified into two principal categories based on cellular origin: carcinomas and sarcomas. Carcinomas account for the vast majority of breast malignancies and originate from the epithelial cells of the terminal ductal-lobular units, which are responsible for milk production. In contrast, sarcomas are rare tumors arising from the stromal components of the breast, including fibroblasts, myofibroblasts, adipose tissue, and vascular structures. Breast sarcomas represent less than 1% of all primary breast cancers, making them clinically uncommon but biologically distinct entities [23,24]. Tumors may also exhibit mixed histological patterns, reflecting clonal heterogeneity and plasticity within breast cancer evolution [22,25].

From a clinical and pathological standpoint, breast cancer is further classified based on invasiveness into three major groups: non-invasive (*in situ*), invasive, and metastatic disease. Non-invasive breast cancers, also referred to as carcinoma *in situ*, remain confined to the ducts or lobules without invasion of the basement membrane. Invasive breast cancers are characterized by tumor cells breaching the basement membrane and infiltrating surrounding stromal tissue, with potential for regional lymph node involvement and distant metastasis. Metastatic breast cancer represents advanced systemic disease in which malignant cells disseminate to distant organs such as bone, liver, lungs, or the brain [15,16].

**Figure 2:**
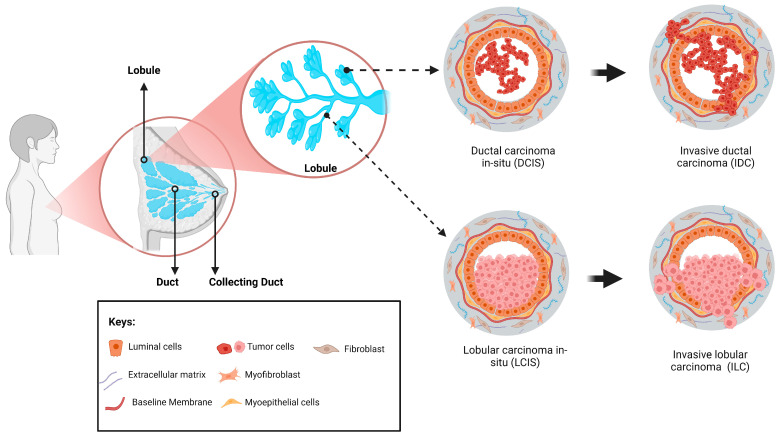
Histological subtypes: ductal and lobular representation of breast cancer. This figure was created by the authors using BioRender.com.

Histologically, the most common invasive subtypes are invasive ductal carcinoma and invasive lobular carcinoma, which together account for the majority of breast cancer cases globally. Recent pathological updates continue to emphasize that despite their different growth patterns and molecular characteristics, these subtypes often share overlapping clinical management strategies while maintaining distinct biological behavior and metastatic patterns [24,26]. Figure 2 illustrates the major histological subtypes of breast cancer, highlighting ductal and lobular differentiation.

In addition to these classical subtypes, recent World Health Organization (WHO)-based classifications recognise a broad spectrum of special histological variants, including mucinous, tubular, papillary, metaplastic, and apocrine carcinomas, each with distinct morphological and clinical features [23,24]. These rare subtypes collectively contribute to the overall heterogeneity of breast cancer and may demonstrate variable prognosis and treatment responses compared to invasive carcinoma of no special type.

### 4.1. Non-Invasive (or In Situ) Breast Cancer

#### Ductal Carcinoma In Situ

Ductal carcinoma *in situ* (DCIS), also referred to as intraductal carcinoma, represents a non-invasive or pre-invasive form of breast cancer arising from epithelial cells confined within the breast ducts without invasion of the basement membrane [22,23]. DCIS is among the most frequently diagnosed forms of breast cancer due to widespread screening programs, particularly mammography. Although non-invasive at diagnosis, DCIS carries a variable risk of progression to invasive breast cancer if left untreated, making early detection and appropriate management clinically important [15,16].

### 4.2. Invasive or Infiltrating Breast Cancer

Invasive breast cancer refers to malignant epithelial tumors that breach the basement membrane and infiltrate surrounding stromal tissue of the breast [22,23]. Once invasive, tumor cells gain the ability to disseminate via lymphatic and haematogenous routes, leading to regional and distant metastases to the bones, brain, liver, and lymph nodes. Invasive breast cancer predominantly affects older women, with incidence increasing with age, particularly after 50 years [16]. Approximately 90% of breast cancers fall within the invasive category, highlighting its dominance in clinical oncology [15].

Invasive carcinomas are broadly divided into the following:

*Invasive Ductal Carcinoma* (IDC). Invasive ductal carcinoma, also referred to as invasive carcinoma of no special type, is the most common histological subtype, accounting for approximately 70–80% of invasive breast cancers worldwide [15,22]. IDC is characterized by malignant epithelial cells forming irregular duct-like structures that invade surrounding breast stroma.

Risk factors for IDC include prolonged estrogen exposure, early menarche (before the age of 12), late menopause (after the age of 55), nulliparity, and genetic predisposition, particularly BReast Cancer gene (BRCA)1/BRCA2 mutations [16,27,28]. IDC typically presents as a palpable breast mass and spreads via lymphatic and vascular routes to regional lymph nodes and distant organs such as bone, liver, lung, and brain [23].

*Invasive Lobular Carcinoma* (ILC). Invasive lobular carcinoma (ILC) is the second most common histological subtype, accounting for approximately 10–15% of invasive breast cancers [24,26]. It is characterized by dyscohesive small, uniform tumor cells infiltrating the stroma in a classic “single-file” pattern due to loss of E-cadherin expression.

ILC typically occurs in older women (i.e., often in the early 60 s) and is often diagnosed at a slightly later age compared to IDC, commonly in the sixth decade of life [26]. It also demonstrates distinct metastatic behaviour, with a higher propensity for spread to unusual sites such as the peritoneum, gastrointestinal tract, and ovaries [24]. Collectively, invasive carcinomas account for the vast majority of breast cancer-related morbidity and mortality globally [15].

### 4.3. Metastatic Breast Cancer

Metastatic breast cancer (stage IV disease) refers to advanced malignancy in which tumor cells have disseminated beyond the breast and regional lymph nodes to distant organs, including bone, liver, lungs, and brain [2,16]. Metastasis may occur early via micrometastatic spread, which can remain clinically undetectable for prolonged periods even after primary tumor removal, contributing to disease recurrence [2].

### 4.4. Molecular Subtypes of Breast Cancer

Contemporary classification systems integrate histopathological features with gene expression profiles and receptor status to define clinically relevant groups that guide prognosis and treatment decisions. The major intrinsic molecular subtypes include luminal (A and B), human epidermal growth factor receptor 2 (HER2)-enriched, and triple-negative/basal-like breast cancer [16,22].

### 4.5. Luminal Breast Cancer Subtypes

Luminal breast cancers are defined by expression of estrogen receptor (ER) and are the most common molecular subtype, accounting for approximately 60–70% of breast cancers in Western populations [15,16,29]. These tumors are characterized by the expression of an estrogen receptor (ER) and/or progesterone receptor (PR), reflecting an origin from luminal epithelial cells of the terminal ductal-lobular unit. They are generally divided into luminal A and luminal B subtypes based on proliferation markers and HER2 status.

### 4.6. Luminal A Subtype

Luminal A tumors are defined by ER-positive and/or PR-positive status, HER2 negativity, and low proliferative activity as measured by Ki-67. They typically demonstrate low histological grade, indolent growth patterns, and favourable long-term prognosis. These tumors are highly responsive to endocrine therapy and are associated with the lowest recurrence rates among all breast cancer subtypes [16,22,29].

### 4.7. Luminal B Subtype

Luminal B tumors are also hormone receptor-positive but demonstrate higher proliferative indices and may be HER2-positive or PR-negative. Compared with Luminal A, Luminal B cancers are biologically more aggressive, with higher recurrence risk and reduced endocrine sensitivity. These tumors often require combined endocrine therapy and chemotherapy, and in HER2-positive cases, targeted anti-HER2 therapy is also indicated [16,24,29]. Collectively, luminal tumors are driven largely by oestrogen signalling pathways, although molecular profiling has revealed significant heterogeneity within this group, suggesting the need for increasingly personalised therapeutic strategies [2].

### 4.8. HER2-Enriched Breast Cancer

HER2-enriched breast cancer is defined by amplification or overexpression of the ERBB2 (HER2) gene and typically lacks expression of ER and PR. This subtype accounts for approximately 10–15% of all breast cancers and is associated with aggressive tumor biology in the absence of targeted therapy [15,22].

HER2 is a transmembrane tyrosine kinase receptor that promotes cell proliferation and survival through activation of downstream signaling pathways such as Phosphatidylinositol-3-kinase/AKT (PI3K/AKT) and Mitogen-Activated Protein Kinase (MAPK). Unlike other receptor tyrosine kinases, HER2 has no known ligand and is activated primarily through dimerization with other ERBB family members, making it constitutively active when overexpressed [16].

The introduction of HER2-targeted therapies, particularly monoclonal antibodies such as trastuzumab and newer antibody–drug conjugates, has dramatically improved survival outcomes in this subgroup, transforming HER2-positive breast cancer from a highly aggressive disease to a largely manageable condition when diagnosed early [2,13].

### 4.9. Triple-Negative Breast Cancer

Triple-negative breast cancer (TNBC) is defined by the absence of ER, PR, and HER2 expression or gene amplification. TNBC accounts for approximately 10–15% of breast cancers and is associated with aggressive clinical behavior, early recurrence, and poorer overall prognosis compared with hormone receptor-positive and HER2-positive subtypes [15,16].

TNBC disproportionately affects younger women, premenopausal patients, and individuals with germline BRCA1/2 mutations. It is also more frequently observed in certain ethnic populations, reflecting both genetic predisposition and structural healthcare disparities [2,24]. The absence of targetable receptors limits endocrine and HER2-directed therapies, making systemic chemotherapy historically the mainstay of treatment.

However, recent advances have expanded the therapeutic landscape of TNBC to include immune checkpoint inhibitors, Poly (ADP-ribose) polymerase (PARP) inhibitors for BRCA-mutated disease, and antibody–drug conjugates, significantly improving outcomes in selected patient groups [13,16,30].

At the molecular level, TNBC is highly heterogeneous and overlaps partially with basal-like breast cancer. However, these classifications are not synonymous, as basal-like status is defined by gene expression profiling rather than receptor status alone [22]. Multi-omic studies have demonstrated that TNBC comprises multiple biologically distinct entities driven by genomic instability, immune microenvironment interactions, and transcriptional reprogramming [23].

The current clinical classification of breast cancer is based primarily on the expression of estrogen receptor (ER), progesterone receptor (PR), HER2, and proliferation markers such as Ki-67. These biomarkers define clinically relevant molecular subtypes that differ substantially in prognosis, patterns of disease progression, and therapeutic response, forming the basis of modern precision oncology and treatment decision-making, as shown in Table 2.

### 4.10. Stages of Breast Cancer

The introduction of prognostic stage groupings that incorporate tumor biology and biomarkers alongside the traditional TNM (Tumor, Node, and Metastasis) classification system has reinforced the importance of molecular characteristics in determining breast cancer prognosis and treatment outcomes [16,38]. The TNM system remains the globally accepted framework for anatomical staging, describing tumor size (T), regional lymph node involvement (N), and presence of distant metastasis (M) [15].

The TNM system classifies breast cancer into stages ranging from 0 to IV, reflecting disease progression from non-invasive lesions to widespread metastatic disease. An increasing stage is associated with a worsening prognosis and reduced survival, highlighting the importance of early detection and intervention [2].

### 4.11. Breast Cancer Development Stages

#### 4.11.1. Stage 0 (Carcinoma *In Situ*/DCIS)

Stage 0 breast cancer refers to non-invasive disease, most commonly ductal carcinoma *in situ* (DCIS), in which abnormal epithelial cells are confined within the ductal or lobular structures without invasion of the basement membrane [22,23,28]. Although non-invasive, DCIS is considered a precursor lesion with variable potential to progress to invasive disease if untreated. Early detection and appropriate management are therefore essential to prevent progression [16].

#### 4.11.2. Stage I (Early Invasive Disease)

**Figure 3:**
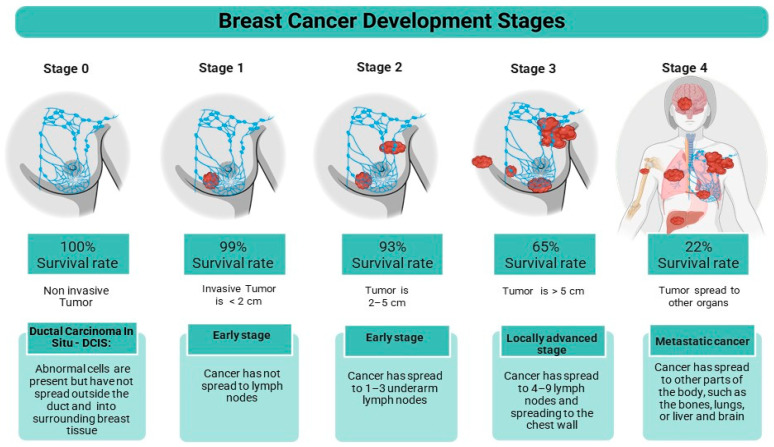
Clinical Representation of Breast Cancer Development Stages. This figure was created by the authors using BioRender.com.

Stage I breast cancer is defined by small invasive tumors (≤2 cm) with either no lymph node involvement or minimal microscopic nodal disease, Figure 3. At this stage, the disease is highly treatable and associated with an excellent prognosis when diagnosed early and managed appropriately [15]. Contemporary population data show high long-term survival rates in high-income settings due to early detection and effective multimodal therapy [2]. Category IA covers tumors that are up to 2 cm in size and do not include any lymph nodes, whereas stage IB represents a tiny clump of cancer cells larger than 0.2 mm discovered in a lymph node [28].

#### 4.11.3. Stage II (Localised Invasive Disease)

Stage II breast cancer includes larger tumors (typically >2 cm) and/or limited regional lymph node involvement. It is subdivided into stages IIA and IIB based on tumor size and nodal status. At this stage, the disease remains potentially curable but requires combined modality treatment including surgery, systemic therapy, and often radiotherapy [16]. Prognosis is still favorable but declines compared with stage I due to increased tumor burden and nodal involvement. However, Phase IIB demonstrates that the cancer has not migrated to the axillary lymphatic nodes, even if it may be larger than 5 cm. At this stage, the survival rate is 93% (Figure 3) [28,39].

#### 4.11.4. Stage III (Locally Advanced Breast Cancer)

Stage III breast cancer is characterized by extensive regional spread, including involvement of multiple axillary lymph nodes or direct extension to the chest wall or skin. It includes inflammatory breast cancer, a particularly aggressive subtype associated with rapid progression and skin involvement [22]. Although no distant metastasis is present, this stage is associated with significantly reduced survival and requires intensive multimodal treatment, including chemotherapy, surgery, and radiotherapy [2]. The overall survival rate at this stage is 65% (Figure 3) [28].

#### 4.11.5. Stage IV (Metastatic Breast Cancer)

Stage IV breast cancer represents advanced disease in which tumor cells have spread to distant organs such as bones, the liver, the lungs, or the brain [28,39]. It is considered incurable with current standard therapies, although treatment can prolong survival and improve quality of life [15,16]. The management of metastatic disease is increasingly personalized, incorporating endocrine therapy, HER2-targeted agents, immunotherapy, PARP inhibitors, and antibody–drug conjugates depending on tumor subtype [2,13].

### 4.12. Screening and Diagnosis of Breast Cancer

Early detection remains the cornerstone of breast cancer control because it reduces disease-specific mortality, enables timely therapeutic intervention, and facilitates individualized treatment planning. Population-based mammographic screening remains the primary imaging modality for breast cancer screening and has been consistently associated with reductions in breast cancer mortality [16]. Ultrasound and magnetic resonance imaging (MRI) serve as important complementary imaging techniques, particularly in women with dense breast tissue, hereditary cancer syndromes, or those at high risk of developing breast cancer, where they improve diagnostic sensitivity beyond mammography alone [40,41,42]. Nevertheless, screening effectiveness varies according to age, breast density, genetic risk, socioeconomic factors, and access to healthcare infrastructure.

The diagnostic evaluation of suspected breast cancer follows the internationally accepted “triple assessment” approach, comprising clinical examination, breast imaging, and histopathological confirmation [16]. Histopathological examination of tissue obtained through core needle biopsy remains the diagnostic gold standard, providing a definitive assessment of tumor histology, grade, receptor status, and molecular subtype, all of which are essential for prognosis and treatment selection [22]. Breast Imaging Reporting and Data System (BI-RADS) categories 4 (suspicious abnormality) and 5 (highly suggestive of malignancy) generally require tissue biopsy for definitive diagnosis. Repeat biopsy is recommended when pathological findings are discordant with clinical or radiological assessment to minimize diagnostic error [42,43]. Rigorous pathological quality assurance remains essential to reduce both false-positive and false-negative diagnoses.

Recent advances in precision oncology have accelerated interest in minimally invasive diagnostic technologies, particularly liquid biopsy. Analysis of circulating tumor DNA (ctDNA), circulating tumor cells (CTCs), circulating tumor RNA, and extracellular vesicles provides valuable insights into tumor evolution, treatment response, minimal residual disease, and mechanisms of therapeutic resistance [44,45]. Although liquid biopsy has demonstrated considerable promise for disease monitoring and precision medicine, several limitations, including assay standardisation, analytical sensitivity in early-stage disease, clinical validation, and cost-effectiveness, currently preclude its routine replacement of tissue biopsy, which remains the diagnostic gold standard [16,45].

Despite substantial technological advances, major disparities persist in the availability and effectiveness of breast cancer screening and diagnostic services worldwide. High-income countries have achieved significant improvements in early detection and survival through organized population-based screening programs and multidisciplinary cancer care. In contrast, screening in many low- and middle-income countries (LMICs) remains predominantly opportunistic rather than systematic because of limited healthcare infrastructure, inadequate funding, workforce shortages, and restricted access to diagnostic technologies [46,47]. Consequently, patients in LMICs are more likely to present with advanced-stage disease, contributing to poorer survival outcomes. Addressing these inequities requires context-specific strategies that strengthen healthcare infrastructure, expand diagnostic capacity, improve workforce training, and increase public awareness to promote timely presentation and early diagnosis.

### 4.13. Breast Cancer and Genetics

Breast cancer is commonly categorized into hereditary, familial (familial clustering), and sporadic forms according to the degree of inherited genetic predisposition and family history [42,48]. Table 3 presents a comparison of the key characteristics of hereditary, familial, and sporadic breast cancer, highlighting differences in genetic predisposition, family history, associated susceptibility genes, age at onset, and estimated contribution to the overall breast cancer burden [42,48]. Genetic susceptibility plays a fundamental role in breast cancer aetiology; however, the majority of cases arise sporadically. Current evidence indicates that approximately 5–10% of breast cancers are attributable to inherited pathogenic germline variants in high- or moderate-penetrance susceptibility genes, including BRCA1, BRCA2, Partner and Localizer of BRCA2 (PALB2), Checkpoint kinase 2 (CHEK2), and tumor protein p53 (TP53) [49,50,51]. A further 15–20% of cases occur in individuals with a positive family history but without an identifiable pathogenic variant, reflecting the combined influence of shared polygenic susceptibility, low-penetrance risk alleles, and environmental or lifestyle factors [50,52]. Consequently, nearly 80–90% of breast cancers are considered sporadic and arise through the accumulation of acquired somatic genetic alterations and environmental exposures [16,42,53].

Hereditary breast cancer results from inherited pathogenic variants in cancer susceptibility genes that confer substantially increased lifetime risks of breast and other malignancies. Individuals carrying high-penetrance pathogenic germline variants are at increased risk of developing breast cancer at younger ages and frequently exhibit distinct clinicopathological and molecular tumor characteristics, reflecting the biological effects of the underlying susceptibility gene [49,50]. Depending on the specific susceptibility gene, lifetime breast cancer risk may exceed 70%, particularly among carriers of pathogenic BRCA1 variants, while BRCA2 and PALB2 pathogenic variants are also associated with substantially elevated lifetime risks approaching or exceeding 50–70%, depending on family history and other modifying factors [42,49,50].

Breast cancer susceptibility genes are commonly classified according to penetrance. High-penetrance genes, including BRCA1, BRCA2, PALB2, TP53, phosphatase and tensin homolog (PTEN), Cadherin-1 (CDH1), and Serine/threonine kinase 11 (STK11), are associated with substantially elevated lifetime breast cancer risks, typically exceeding 30–60%, with certain variants particularly pathogenic BRCA1 mutations conferring risks approaching or exceeding 70–80% [42,50,53]. Moderate-penetrance genes, including ATM, CHEK2, BRCA1-Associated Ring Domain 1 (BARD1), RAD51C, RAD51D, and BRCA1 interaction protein C-terminal helicase 1 (BRIP1), are generally associated with approximately two- to four-fold increases in relative breast cancer risk. However, absolute lifetime risk varies considerably depending on factors such as family history, ancestry, and polygenic risk background, with some variants (e.g., truncating CHEK2 mutations or specific ATM variants) conferring higher risk estimates in certain populations [42,50,53].

Inherited pathogenic genetic alterations promote breast carcinogenesis through disruption of critical cellular pathways, including DNA damage repair, cell-cycle regulation, apoptosis, and maintenance of genomic stability. Loss-of-function mutations in tumor suppressor genes (e.g., BRCA1, BRCA2, TP53, PTEN) compromise DNA repair fidelity and genomic integrity, while activation of oncogenic signaling pathways enhances cellular proliferation and tumor progression. In contrast to somatic alterations, germline pathogenic variants are typically inherited in an autosomal dominant manner with variable penetrance, enabling vertical transmission across generations and forming the genetic basis of hereditary breast cancer syndromes [42].

In addition to inherited susceptibility genes, several molecular biomarkers are routinely assessed in breast cancer to inform prognostic assessment and therapeutic decision-making. Key biomarkers include oestrogen receptor (ER; encoded by ESR1), progesterone receptor (PR; PGR), human epidermal growth factor receptor 2 (HER2; ERBB2), and the proliferation marker Ki-67 (MKI67), which together underpin contemporary molecular classification systems and the principles of precision oncology. Clinically, biomarkers are broadly classified as prognostic or predictive: prognostic biomarkers provide information on disease outcome independent of therapy, whereas predictive biomarkers identify patients who are more likely to benefit from specific systemic or targeted treatments. Increasingly, multigene expression signatures and comprehensive genomic profiling are being integrated into clinical practice to refine risk stratification and individualise treatment selection beyond conventional immunohistochemical markers [16,54]. Table 4A provides an overview of hereditary susceptibility genes associated with breast cancer predisposition, while Table 4B summarises clinically relevant biomarkers with established prognostic and predictive utility in breast cancer management, including their roles in diagnosis, risk stratification, and therapeutic decision-making.

Breast cancer diagnostic biomarkers are increasingly being classified according to their biological source, including serum, urine, and tissue, enabling more precise approaches to diagnosis, prognosis, and treatment response monitoring. While established tissue-based biomarkers such as oestrogen receptor (ER), progesterone receptor (PR), and human epidermal growth factor receptor 2 (HER2) remain the gold standard for tumor classification, emerging liquid biopsy biomarkers provide minimally invasive alternatives for real-time disease assessment. These include circulating tumor DNA (ctDNA), circulating tumor cells (CTCs), circulating tumor RNA, and extracellular vesicles, which have demonstrated increasing clinical utility in early detection, monitoring of treatment response, and identification of resistance mechanisms in breast cancer [16,55,56]. In addition, urine-based biomarker assays are being explored as non-invasive tools for longitudinal disease monitoring, although their clinical validation remains limited. Collectively, liquid biopsy approaches offer a dynamic reflection of tumor heterogeneity and evolution, supporting more personalised diagnostic and therapeutic strategies in breast cancer management [57,58]. Table 5 summarises key diagnostic biomarkers in breast cancer, their biological sources, and associated analytical techniques and clinical applications.

### 4.14. Precision Medicine for Breast Cancer

The transition from conventional “one-size-fits-all” treatment approaches to precision medicine has fundamentally reshaped breast cancer management by enabling more individualised risk stratification and therapy selection. This paradigm integrates multi-omics data, including genomic, transcriptomic, epigenomic, and metabolomic profiles, to refine tumor classification, predict therapeutic response, and monitor disease progression [16,79]. In clinical practice, molecular subtyping and multigene expression assays have further improved prognostic accuracy and supported more tailored treatment decision-making, thereby enhancing clinical outcomes across different breast cancer subtypes [80,81].

Beyond conventional tissue biopsy, liquid biopsy technologies, including circulating tumor DNA (ctDNA), circulating tumor cells (CTCs), and extracellular vesicles, are increasingly being incorporated into clinical oncology to capture tumor heterogeneity and provide real-time insights into disease evolution. This minimally invasive approach supports longitudinal monitoring of treatment response and resistance, addressing key limitations associated with spatial and temporal heterogeneity in traditional tissue sampling [44,45]. Despite these advances, the implementation of precision medicine remains uneven globally. High-income countries have rapidly integrated molecular diagnostics into routine cancer care, whereas low- and middle-income countries continue to face substantial barriers related to cost, limited access to genomic sequencing technologies, and inadequate bioinformatics infrastructure [16,47]. Bridging this gap will require scalable, cost-effective biomarker platforms and the integration of multi-omics approaches into context-appropriate healthcare systems to ensure equitable access to precision oncology.

### 4.15. Treatment and Management of Breast Cancer

Several therapeutic strategies have been developed over time to treat breast cancer and reduce disease-related mortality, including locoregional and systemic approaches such as surgery, radiotherapy, endocrine therapy, chemotherapy, targeted agents, and immunotherapy, with current treatment paradigms increasingly informed by molecular subtyping and tumor biology [1,16]. However, increasing recognition of molecular and clinical heterogeneity in breast cancer has driven the evolution of more individualised and stratified treatment approaches over recent decades [82,83]. Tumor heterogeneity in breast cancer is characterised by spatial, temporal, and clonal diversity that drives disease progression, metastasis, and therapeutic resistance. Recent advances in multi-omics profiling, single-cell sequencing, and liquid biopsy technologies have demonstrated that tumors evolve dynamically under treatment pressure, leading to the emergence of resistant subclonal populations [82,83,84]. These findings have redefined cancer as a continuously evolving ecosystem rather than a static disease, reinforcing the need for longitudinal molecular monitoring using minimally invasive approaches such as circulating tumor DNA analysis [85,86].

In this review, breast cancer therapies are broadly categorised into three main groups: conventional treatment approaches, including surgery, radiotherapy, chemotherapy, and hormonal (endocrine) therapy; targeted and immune-based therapies, which form a central component of contemporary clinical management; alongside novel and emerging therapeutic strategies, including molecularly guided interventions, immunotherapies, antibody–drug conjugates (ADCs), and other advanced platforms aiming to improve survival outcomes while reducing treatment-related toxicity [1,13,16,80].

### 4.16. Conventional Treatment

#### 4.16.1. Surgery

Surgery remains the cornerstone of breast cancer (BC) management and is the primary treatment modality for most patients with early-stage disease. Surgical treatment generally consists of either breast-conserving surgery (lumpectomy) or mastectomy, accompanied by sentinel lymph node biopsy or axillary lymph node dissection according to tumor stage and nodal involvement [1,87]. Increasing adoption of breast-conserving surgery has been facilitated by advances in screening, early diagnosis, oncoplastic techniques, and adjuvant radiotherapy, resulting in survival outcomes comparable to those achieved with mastectomy in appropriately selected patients [1,87].

However, substantial disparities in surgical management persist globally. In high-income countries, organised screening programmes, multidisciplinary care, and improved access to radiotherapy have contributed to increased utilisation of breast-conserving surgery. Conversely, mastectomy remains the predominant surgical approach across many low- and middle-income countries (LMICs), where delayed presentation, advanced-stage disease at diagnosis, limited radiotherapy availability, inadequate surgical infrastructure, workforce shortages, and fragmented healthcare systems continue to restrict treatment options [47,87,88].

In many African settings, treatment decisions are additionally influenced by socioeconomic constraints, high out-of-pocket healthcare costs, prolonged waiting times, surgeon recommendation, cultural beliefs, stigma associated with breast cancer, and limited access to postoperative surveillance and reconstructive services [87,89,90].

Persistent inequities in access to breast cancer surgery contribute significantly to poorer survival outcomes in LMICs and underserved populations. Addressing these disparities will require investment in surgical infrastructure, expansion of multidisciplinary oncology services, improved workforce capacity, equitable access to radiotherapy and reconstruction, and implementation of health-system interventions that promote timely diagnosis and treatment [91,92,93].

#### 4.16.2. Radiotherapy

Radiotherapy is a fundamental component of multimodal breast cancer treatment and is routinely administered following breast-conserving surgery, as well as in selected patients after mastectomy who are at high risk of locoregional recurrence. Adjuvant radiotherapy has been shown to significantly reduce local recurrence and improve both disease-free and overall survival, making it a standard of care for many patients with early-stage and locally advanced breast cancer [1,94].

Despite its established clinical benefits, access to radiotherapy remains highly inequitable worldwide. While high-income countries have increasingly adopted advanced radiotherapy techniques and hypofractionated treatment schedules that improve efficiency and patient convenience, many low- and middle-income countries (LMICs) continue to experience substantial shortages of radiotherapy infrastructure, trained personnel, and maintenance capacity, resulting in treatment delays, reduced utilisation of breast-conserving surgery, and poorer clinical outcomes [47,92,95].

The disparity is particularly pronounced across Africa, where radiotherapy services remain critically under-resourced. Although some expansion in radiotherapy capacity has occurred in recent years, access remains concentrated in a limited number of countries, with many patients travelling long distances or experiencing prolonged waiting times for treatment. Consequently, inadequate radiotherapy availability continues to influence surgical decision-making, often favouring mastectomy over breast-conserving surgery in settings where postoperative radiotherapy cannot be reliably delivered [47,94,96]. Addressing these disparities will require sustained investment in radiotherapy infrastructure, workforce development, regional cancer centres, and equitable implementation of national cancer control programmes to improve access and breast cancer outcomes across LMICs [47,95]. 

#### 4.16.3. Chemotherapy

Chemotherapy remains a central component of systemic breast cancer treatment, particularly for biologically aggressive subtypes (such as triple-negative and HER2-positive disease) and for patients with locally advanced or metastatic presentations [1,97]. It is commonly administered in either the neoadjuvant or adjuvant setting, with standard regimens frequently incorporating anthracyclines and taxanes. These regimens aim to eliminate micrometastatic disease, improve disease-free and overall survival, and, in the neoadjuvant context, achieve tumor downstaging to increase the feasibility of breast-conserving surgery [1,98].

Despite its clinical effectiveness, the use of chemotherapy is limited by inter-patient variability in treatment response, cumulative toxicities (including cardiotoxicity, neuropathy, and myelosuppression), and challenges in delivering optimal supportive care. These limitations are particularly pronounced in low- and middle-income countries (LMICs), where late-stage presentation, limited access to pathology services, drug supply constraints, and inadequate supportive care infrastructure can significantly reduce treatment effectiveness [47,92,95].

Consequently, the management of chemotherapy-related toxicities, alongside improved risk stratification and biomarker-driven treatment selection, remains essential to maximising therapeutic benefit while minimising harm, particularly in resource-constrained settings [1,97].

#### 4.16.4. Hormonal Therapy

Endocrine therapy remains a cornerstone in the management of hormone receptor-positive breast cancer and has substantially improved long-term survival outcomes. Common agents, including selective oestrogen receptor modulators (e.g., tamoxifen) and aromatase inhibitors, function by inhibiting oestrogen-driven signalling pathways that are essential for tumor proliferation and progression [1,97]. The effectiveness of endocrine therapy is critically dependent on accurate determination of hormone receptor status (oestrogen and progesterone receptors), underscoring the importance of reliable pathology and immunohistochemistry infrastructure to guide appropriate patient selection [47,92].

However, in many low- and middle-income countries (LMICs), limited access to standardised immunohistochemical testing, delayed pathology turnaround times, and shortages of trained personnel continue to hinder optimal treatment stratification and contribute to suboptimal therapeutic outcomes [47,95]. These diagnostic gaps remain a key barrier to equitable delivery of endocrine therapy in resource-constrained settings.

Despite its effectiveness, resistance to endocrine therapy remains a major clinical challenge. Both intrinsic and acquired resistance are driven by tumor heterogeneity, activation of alternative signalling pathways, and genomic evolution under therapeutic pressure. These mechanisms have driven the development of combination strategies and targeted agents (such as CDK4/6 inhibitors) to enhance endocrine responsiveness and delay disease progression [1,97]. Consequently, contemporary breast cancer management is increasingly shifting towards biomarker-driven and combination therapeutic approaches to overcome resistance while maintaining treatment tolerability.

#### 4.16.5. Proteolysis-Targeting Chimeras (PROTACs)

Proteolysis-targeting chimeras (PROTACs) represent an emerging therapeutic strategy that enables selective degradation of disease-relevant proteins via the ubiquitin–proteasome system (UPS), the cell’s intrinsic protein disposal machinery [99,100]. Unlike conventional small-molecule inhibitors that merely suppress protein function, PROTACs employ bifunctional molecules to recruit target proteins to the UPS, leading to their degradation. This mechanism offers several advantages, including improved selectivity, catalytic activity, and the ability to target proteins that have historically been considered undruggable [101]. Recent advances in targeted protein degradation have further accelerated PROTAC development, with multiple candidates entering clinical trials and demonstrating early therapeutic promise in oncology settings, reinforcing the modality’s translational potential [100].

The FDA approval of vepdegestrant (ARV-471; Veppanu) for patients with ESR1-mutated, ER-positive/HER2-negative advanced or metastatic breast cancer represents a landmark achievement in the clinical translation of targeted protein degradation. As the first PROTAC-based therapy to receive regulatory approval, vepdegestrant validated the therapeutic potential of the PROTAC platform by demonstrating superior progression-free survival compared with fulvestrant in the phase III VERITAC-2 trial, thereby establishing targeted protein degradation as a viable therapeutic modality in precision oncology [102,103].

#### 4.16.6. Radioligand Therapy

Radioligand therapy has been incorporated as an emerging precision oncology approach in metastatic breast cancer, reflecting growing evidence of its therapeutic potential and favorable safety profile [104,105]. HER2-targeted radioligand imaging is expected to significantly advance the diagnostic landscape in HER2-positive breast cancer by enabling more precise, non-invasive assessment of receptor expression and treatment response, including spatial and temporal tumor heterogeneity in vivo [104,106]. This approach may be particularly valuable in evaluating emerging targeted therapies, including antibody–drug conjugates (ADCs), by improving early response prediction and supporting treatment stratification and personalised therapeutic decision-making [104,105,107].

### 4.17. Targeted and Immune-Based Therapies

#### 4.17.1. Targeted Therapy

The advent of targeted therapies has significantly transformed the management of breast cancer by enabling biologically driven, subtype-specific treatment strategies [1,97,108]. In HER2-positive breast cancer, which is historically associated with poorer prognosis, the introduction of anti-HER2 monoclonal antibodies such as trastuzumab and pertuzumab, alongside antibody–drug conjugates and tyrosine kinase inhibitors, has markedly improved survival outcomes in both early-stage and metastatic disease [97,98,109,110].

In hormone receptor-positive, HER2-negative breast cancer, cyclin-dependent kinase 4/6 (CDK4/6) inhibitors, including palbociclib, ribociclib, and abemaciclib, have become integral components of standard care. When combined with endocrine therapy, these agents significantly prolong progression-free survival and delay disease progression [1,108,110]. In addition, targeting downstream signalling pathways such as PI3K/AKT/mTOR has broadened therapeutic strategies for endocrine-resistant and molecularly defined breast cancer subtypes; however, therapeutic efficacy is largely confined to patients with specific genomic alterations, highlighting persistent challenges in biomarker selection and intratumoral heterogeneity [1,97,111].

Despite these advances, access to targeted therapies remains highly unequal globally. In many low- and middle-income countries (LMICs), high treatment costs, limited availability of molecular diagnostic testing, inadequate genomic profiling infrastructure, and health system constraints continue to restrict the widespread implementation of precision oncology approaches [47,95]. These disparities highlight the ongoing challenge of translating advances in targeted therapy into equitable clinical benefit worldwide.

#### 4.17.2. Immunotherapy

Immunotherapy has emerged as a promising therapeutic approach in breast cancer management, particularly in triple-negative breast cancer (TNBC), which is associated with aggressive clinical behaviour and limited targeted treatment options [30,97]. Immune checkpoint inhibition targeting the PD-1/PD-L1 axis, in combination with chemotherapy, has demonstrated improved progression-free and overall survival in selected patients with PD-L1-positive tumors, establishing immunotherapy as a standard component of treatment in this subgroup [1,30].

Ongoing clinical trials are evaluating the role of immunotherapy in earlier disease settings and in combination with other targeted agents, with the aim of overcoming resistance mechanisms and enhancing response durability across additional breast cancer subtypes [97]. However, despite these advances, the clinical utility of immunotherapy remains limited by variable response rates, the absence of universally reliable predictive biomarkers, and the potential for immune-related adverse events affecting multiple organ systems [1,112,113,114]. These challenges, combined with high treatment costs and limited access to biomarker testing, further restrict widespread implementation in low- and middle-income countries [47,95].

#### 4.17.3. Antibody–Drug Conjugates

Antibody–drug conjugates (ADCs) represent a major advancement in breast cancer therapy by combining tumor-targeting monoclonal antibodies with highly potent cytotoxic payloads, thereby enhancing tumor specificity while reducing systemic toxicity [97,98,115]. In HER2-positive disease, agents such as trastuzumab emtansine (T-DM1) and trastuzumab deruxtecan (T-DXd) have demonstrated substantial improvements in progression-free and overall survival, while T-DXd has additionally shown efficacy in HER2-low breast cancer, expanding the therapeutic landscape beyond traditional HER2 classification [1,98,116,117].

Sacituzumab govitecan has demonstrated significant improvements in progression-free and overall survival compared with standard chemotherapy in heavily pretreated metastatic triple-negative breast cancer, as shown in the phase III ASCENT trial [97,118].

### 4.18. Emerging and Precision Approaches Employed in Breast Cancer Management and Treatment

#### 4.18.1. PARP Inhibitors

Poly (ADP-ribose) polymerase (PARP) inhibitors represent a key advancement in precision oncology, particularly for patients with germline or somatic BRCA1/2 mutations and homologous recombination deficiency (HRD) [97,119,120]. These agents exploit the principle of synthetic lethality, selectively inducing tumor cell death in DNA repair–deficient cancer cells while sparing normal tissues [121].

PARP inhibitors, particularly olaparib, have demonstrated clinical benefit in both early and metastatic HER2-negative breast cancer with germline BRCA mutations, as shown in the OlympiA and OlympiAD trials, with long-term follow-up confirming sustained efficacy and survival benefit in selected patient populations [67,119].

Agents such as olaparib and talazoparib have demonstrated improved progression-free survival in both early-stage high-risk and metastatic HER2-negative breast cancer, reinforcing their role in personalised treatment strategies [67,122]. However, the emergence of resistance often driven by restoration of homologous recombination repair or secondary BRCA reversion mutations remains a major limitation and supports ongoing investigation into combination strategies involving PARP inhibitors with chemotherapy, immunotherapy, or targeted agents [1,97].

#### 4.18.2. Molecular Precision Medicine Approach

Advances in genomic and transcriptomic profiling have transformed breast cancer management by enabling precision medicine approaches that tailor treatment according to the molecular characteristics of individual tumors, moving beyond the traditional one-size-fits-all treatment paradigm [1,123]. By accounting for both intertumor and intratumor heterogeneity, these approaches facilitate more accurate risk stratification, optimise therapeutic efficacy, and minimise unnecessary treatment-related toxicity. Multigene expression assays, including Oncotype DX, MammaPrint, Prosigna, and EndoPredict, have become valuable tools for estimating recurrence risk and guiding adjuvant chemotherapy decisions in patients with early-stage hormone receptor-positive, HER2-negative breast cancer [61,124]. Furthermore, advances in next-generation sequencing (NGS) have enabled the identification of clinically actionable genomic alterations, including mutations in PIK3CA, AKT1, ESR1, and BRCA1/2, facilitating the selection of targeted therapies and supporting increasingly personalised treatment strategies [80,125].

#### 4.18.3. Epigenetic Therapies

In both the initiation and progression of breast cancer, epigenetic dysfunction plays a significant role, as well as in resistance to therapy [126]. Recently developed epigenetic therapies targeting reversible modifications in chromatin function, such as DNA methyltransferases and histone deacetylases, have emerged with the goal of re-establishing a normal epigenetic mark and restoring responsiveness to established therapies. Although most epigenetic drugs are in early-phase trials, available preclinical and translational literature shows promise, especially in treatment-refractory aggressive forms of breast cancer. Further characterisation of epigenetic markers will be important in guiding integration into therapy [127,128].

In both the initiation and progression of breast cancer, epigenetic dysregulation plays a critical role, contributing not only to tumor development but also to therapeutic resistance [126,129]. Aberrant DNA methylation, histone modification, and chromatin remodelling alter gene expression patterns without changing the underlying DNA sequence, thereby promoting oncogenic signalling and treatment resistance. These reversible modifications have led to growing interest in epigenetic therapies aimed at restoring normal chromatin architecture and resensitising tumors to conventional and targeted treatments.

Epigenetic agents, including DNA methyltransferase inhibitors (e.g., azacitidine and decitabine) and histone deacetylase (HDAC) inhibitors (e.g., vorinostat and entinostat), are currently under investigation in breast cancer, particularly in treatment-resistant and aggressive subtypes such as triple-negative breast cancer (T-NBC) [124,130]. Although most epigenetic therapies remain in early-phase clinical development, preclinical and translational studies suggest potential synergistic effects when combined with chemotherapy, endocrine therapy, or immunotherapy, particularly in reversing drug resistance phenotypes.

Further characterisation of epigenetic biomarkers, including DNA methylation signatures, histone modification patterns, and chromatin accessibility profiles, will be essential for identifying patients most likely to benefit from these emerging strategies and for guiding future integration into precision oncology frameworks [124,130,131].

#### 4.18.4. Tumor Microenvironment Targeted Approach

The tumor microenvironment (TME) plays a critical role in breast cancer progression and therapeutic responsiveness. Consequently, targeting the TME has emerged as a promising therapeutic strategy, while TME-associated features increasingly serve as both prognostic and predictive biomarkers for guiding precision oncology [132,133]. Emerging therapeutic strategies targeting the tumor microenvironment (TME) seek to disrupt the dynamic interactions between tumor cells and the surrounding stromal, vascular, and immune compartments that drive tumor growth, invasion, metastasis, and therapeutic resistance. Current TME-directed approaches include targeting cancer-associated fibroblasts (CAFs), tumor angiogenesis, tumor-associated macrophages (TAMs), myeloid-derived suppressor cells (MDSCs), regulatory T cells (Tregs), and immune checkpoint-mediated T-cell exhaustion. By remodelling the immunosuppressive tumor milieu, these strategies aim to enhance antitumor immunity, improve treatment responsiveness, and overcome resistance to conventional therapies, targeted agents, and immunotherapies [133,134].

Anti-angiogenic agents, macrophage-directed therapies, and immune-modulating strategies represent promising therapeutic modalities that may enhance antitumor immunity and improve the efficacy of existing systemic treatments [133,134]. Preclinical and early clinical studies further suggest that targeting the TME may overcome therapeutic resistance to chemotherapy, targeted therapies, endocrine therapy, and immunotherapy by reshaping the immunosuppressive tumor milieu and enhancing treatment responsiveness [133,134,135].

#### 4.18.5. Personalized Vaccines and mRNA-Based Platforms

Recent advances in immunotherapy have renewed interest in the development of personalised cancer vaccines, particularly messenger RNA (mRNA)-based platforms designed to elicit tumor-specific immune responses [135,136,137]. These vaccines exploit patient-specific tumor neoantigens identified through genomic sequencing to induce durable and highly specific cytotoxic T-cell responses while minimising off-target toxicity.

Although personalised cancer vaccines remain in the early stages of clinical development for breast cancer, early-phase clinical and preclinical studies have demonstrated encouraging safety, immunogenicity, and preliminary antitumor activity, supporting their potential integration with immune checkpoint inhibitors, chemotherapy, and other targeted therapies to enhance treatment outcomes [136,137]. However, challenges related to neoantigen identification, vaccine manufacturing, tumor heterogeneity, immune escape mechanisms, and cost constraints continue to limit widespread clinical implementation.

#### 4.18.6. Nanotechnology and Advanced Drug-Delivery Systems

Recent advances in nanotechnology have accelerated the development of precision drug delivery systems capable of enhancing tumor-specific drug accumulation, improving therapeutic efficacy, and reducing systemic toxicity, thereby supporting more personalised approaches to breast cancer treatment [138]. Nanocarrier-based platforms, including liposomes, polymeric nanoparticles, micelles, dendrimers, and extracellular vesicles (particularly exosome-based delivery systems), have demonstrated improved pharmacokinetic profiles, enhanced tumor accumulation through the enhanced permeability and retention (EPR) effect, and reduced off-target toxicity compared with conventional chemotherapy [138,139].

As translational research advances, nanomedicine is increasingly positioned as a key component of future breast cancer therapeutics, offering the potential to enhance tumor-specific drug delivery and improve treatment efficacy while reducing systemic toxicity. Table 6 summarises conventional cytotoxic, targeted, immunotherapeutic, and emerging treatment strategies in breast cancer management.

#### 4.18.7. Combination Therapy

Single-agent therapy approaches are no longer sufficient for achieving long-term disease control due to the intrinsic heterogeneity of breast cancer, driven by both intertumoral and intratumoral genomic variation. Combination therapy has therefore become a central strategy in contemporary breast cancer management, aiming to enhance therapeutic efficacy, overcome drug resistance, and reduce the risk of disease recurrence [61,151,152]. To target multiple oncogenic signalling pathways simultaneously, combination regimens commonly integrate agents with complementary mechanisms of action, including immunotherapy with cytotoxic chemotherapy, endocrine therapy with cyclin-dependent kinase (CDK4/6) inhibitors, and targeted therapy with antibody–drug conjugates or other molecularly guided agents [61,152,153].

Treatment paradigms for hormone receptor-positive, HER2-negative breast cancer have been substantially transformed by the combination of endocrine therapy with CDK4/6 inhibitors (palbociclib, ribociclib, and abemaciclib). Contemporary evidence from updated systematic reviews, umbrella reviews, and clinical guidelines confirms that these combinations significantly improve progression-free survival and overall survival in both early and metastatic settings by concurrently inhibiting estrogen receptor signalling and cell-cycle progression [61,154,155]. Similarly, patients with pathway-specific alterations, particularly PIK3CA mutations, derive clinical benefit from combining endocrine therapy with targeted inhibition of the PI3K/AKT/mTOR signalling pathway, highlighting the importance of molecular stratification in guiding precision treatment strategies and overcoming endocrine resistance in HR-positive advanced breast cancer [54,156,157].

Due to the synergistic suppression of HER2 signaling and increased immune-mediated cytotoxicity, dual HER2 blockade utilizing monoclonal antibodies like trastuzumab and pertuzumab in conjunction with chemotherapy has become the standard of care for HER2-positive breast cancer [1,158]. Trastuzumab deruxtecan is one of the antibody–drug conjugates (ADCs) that have been incorporated into combination regimens to increase therapy options. These ADCs have shown efficacy even in HER2-low disease and have challenged conventional subtype boundaries [1,159]. Combination therapies have been especially helpful for triple-negative breast cancer (TNBC), which is marked by aggressive behavior and few therapeutic targets. In certain patients with PD-L1-positive malignancies, the addition of immune checkpoint drugs that target the PD-1/PD-L1 axis to chemotherapy has improved outcomes, underscoring the significance of immunogenic cell death in boosting anticancer immune responses [160,161]. Multi-agent combos comprising immunotherapy, PARP inhibitors, and ADCs are still being studied in ongoing clinical trials to boost therapeutic response and get around intrinsic resistance mechanisms.

### 4.19. Drug Repurposing

Drug repurposing (also referred to as drug repositioning) involves the use of existing approved agents for new therapeutic indications and has become an important strategy in breast cancer management due to established safety profiles, reduced development timelines, and cost-effectiveness [162,163]. This approach is particularly relevant in resource-limited settings where access to novel targeted therapies may be restricted, thereby offering a practical pathway to expand treatment availability and improve equity in cancer care [162,164,165]. Rather than broadly categorizing drugs, their clinical utility in breast cancer is best understood according to treatment intent, including curative/adjuvant therapy, metastatic disease management, palliative care, and risk reduction.

Classical cytotoxic chemotherapies, including cyclophosphamide, doxorubicin, taxanes (paclitaxel and docetaxel), and antimetabolites (fluorouracil, capecitabine, methotrexate, and gemcitabine), remain central to breast cancer management and continue to be widely incorporated into contemporary treatment regimens [163,164]. Among these, gemcitabine is primarily used in metastatic or recurrent breast cancer, typically as part of combination regimens for advanced disease [164,166].

Vinblastine is not a first-line agent in breast cancer but may be considered in selected cases for palliative management of advanced or refractory disease, typically as part of later-line cytotoxic sequencing where treatment goals are symptom control and disease stabilization rather than cure [167,168]. In contemporary metastatic breast cancer treatment algorithms, vinblastine is grouped among older microtubule inhibitors that may be used in heavily pretreated patients when standard regimens (anthracyclines, taxanes, capecitabine, eribulin, gemcitabine-based combinations) have been exhausted or are unsuitable, particularly in the palliative setting of metastatic or refractory disease [60,166].

Endocrine therapy represents another major treatment category. Selective estrogen receptor modulators (SERMs), such as tamoxifen, remain important in adjuvant therapy and risk reduction strategies within hormone receptor–positive breast cancer management frameworks [60,157]. In contrast, raloxifene is not used for active breast cancer treatment but is applied in primary prevention of hormone receptor–positive breast cancer in high-risk postmenopausal women, highlighting its role as a chemopreventive rather than therapeutic agent [60,169]. Aromatase inhibitors, including letrozole, anastrozole, and exemestane, remain standard components of endocrine therapy for postmenopausal hormone receptor–positive breast cancer in both adjuvant and metastatic settings [60,61,157].

Selective estrogen receptor degraders (SERDs), such as fulvestrant, are used in endocrine-resistant metastatic breast cancer [60,157]. Similarly, CDK4/6 inhibitors such as palbociclib and mTOR inhibitors such as everolimus are incorporated into the management of advanced hormone receptor–positive breast cancer due to their effects on cell cycle progression and proliferative signaling pathways [60,61,166].

Beyond standard oncology agents, several non-oncologic drugs have demonstrated potential anticancer effects. Metformin exhibits antitumor activity through activation of AMP-activated protein kinase (AMPK) and inhibition of the mammalian target of rapamycin (mTOR) pathway, although current clinical evidence has not established a definitive survival benefit in unselected breast cancer populations [61,170,171].

Aspirin and other non-steroidal anti-inflammatory drugs (NSAIDs) have been investigated for their potential anticancer effects through cyclooxygenase inhibition, reduced prostaglandin synthesis, and modulation of the tumor microenvironment. Although observational studies and recent meta-analyses suggest that post-diagnostic aspirin use may reduce breast cancer-specific mortality, evidence remains insufficient to support its routine use in clinical practice [60,172].

Antimalarial agents such as hydroxychloroquine and chloroquine have been repurposed because of their ability to inhibit autophagy, a survival mechanism exploited by cancer cells under therapeutic stress. Preclinical and early-phase clinical studies suggest that autophagy inhibition may enhance the sensitivity of breast cancer cells to chemotherapy, particularly in treatment-resistant disease; however, these agents remain investigational and are not recommended as standard breast cancer therapy [60,173,174,175]. Statins, originally used for the treatment of hypercholesterolemia, have also been investigated for their potential anticancer effects through inhibition of the mevalonate pathway, disruption of tumor cell signaling, and modulation of tumor proliferation. Although observational studies and recent systematic reviews suggest a possible association with improved breast cancer outcomes, current evidence is insufficient to support their routine use as anticancer agents outside clinical trials [60,176,177].

There is increasing evidence that repurposed agents may also reduce the risk of breast cancer recurrence, although findings remain heterogeneous and largely derived from observational or preclinical studies [60,176]. Despite these potential benefits, variable trial results, inconsistent patient selection, and the absence of standardized dosing regimens continue to limit the clinical integration of repurposed drugs, underscoring the need for well-designed randomized controlled trials to confirm therapeutic efficacy and safety in defined breast cancer subgroups [60,157].

### 4.20. Treatment Challenges

Treatment resistance continues to be a critical obstacle limiting long-term survival and sustainable responses despite significant advancements in breast cancer therapy. Resistance results from a complex interaction of genetic mutations, altered signaling pathways, tumor heterogeneity, drug metabolism variations, and microenvironmental influences [178,179]. Resistance can be intrinsic (existing prior to treatment) or acquired (developing during therapy) and is driven by mechanisms such as drug efflux, epigenetic modifications, cancer stem cell plasticity, metabolic reprogramming, immune evasion, and stromal remodeling within the tumor microenvironment [110,179]. In addition to undermining the effectiveness of current therapies, this complex nature calls for the continuous development of innovative treatment strategies, including combination therapies, biomarker-guided precision medicine, and next-generation targeted therapeutics to overcome resistance and improve patient outcomes [179,180].

#### 4.20.1. Drug Resistance

Changes at the drug target, downstream signaling reprogramming, compensatory pathway activation, and enhanced DNA repair mechanisms are well-established contributors to therapeutic resistance in breast cancer. For instance, the upregulation of alternative signaling pathways such as PI3K/AKT/mTOR and MAPK can bypass estrogen dependence in hormone receptor-positive breast cancer, thereby reducing the efficacy of endocrine therapies, including aromatase inhibitors and selective estrogen receptor degraders (SERDs) [178,179]. Clinical resistance to anti-estrogen therapies, such as fulvestrant and tamoxifen, is also frequently associated with activating mutations in the ESR1 gene, which encodes the estrogen receptor. These mutations stabilize the receptor in a constitutively active conformation, enabling ligand-independent signaling and sustained tumor growth despite estrogen deprivation [97]. The increasing detection of ESR1 mutations in metastatic breast cancers following prolonged endocrine therapy further underscores their critical role in acquired resistance, disease progression, and therapeutic failure [178].

#### 4.20.2. Cytochrome p450 Enzymes

Endocrine therapy is made more difficult by cytochrome P450 enzymes, especially CYP2D6, which affects drug metabolism. The common anti-estrogen tamoxifen needs to be converted by CYP2D6 into its active metabolite, 4-hydroxytamoxifen. CYP2D6 polymorphisms can decrease the production of active metabolites, which can result in less effective treatments and worse clinical outcomes [181,182,183]. The metabolism of taxanes and other chemotherapeutic medicines is also impacted by variations in cytochrome P450 activity; for example, CYP3A4 and CYP2C8 polymorphisms have been connected to lower plasma drug concentrations, which contribute to chemotherapy resistance [181]. The necessity of pharmacogenomic profiling to tailor treatment and steer clear of ineffective regimens is highlighted by these metabolic variations.

#### 4.20.3. BRCA1/2 Mutations

**Figure 4:**
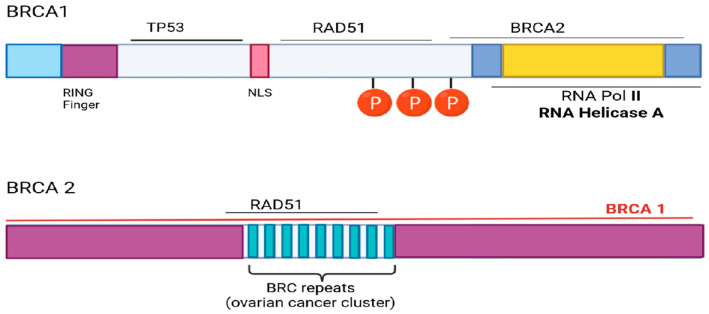
Schematic representation of BRCA1 and BRCA2 genes. This figure was adapted from [186] but recreated by the authors using BioRender.com.

**Figure 5:**
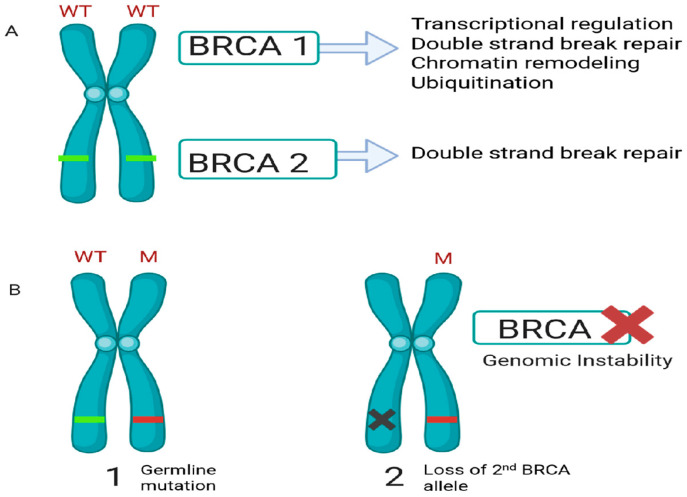
(**A**) Schematic representation of BRCA1 and BRCA2 functions. (**B**) Loss of second *BRCA* allele in a *BRCA* mutation carrier. This figure was adapted from [186] but recreated by the authors using BioRender.com. Key: Blue chromosome- Homologous chromosomes. WT (red text)—Wild-type (normal, functional) BRCA allele; M (red text)—Mutant BRCA allele; Green band—Functional (normal) BRCA gene; Red band—Mutated BRCA gene; Black X on gene—Loss/inactivation of the remaining normal BRCA allele (second hit); Red X over BRCA—Complete loss of BRCA function.

BRCA1/2 mutations, especially in triple-negative and HER2-negative breast tumors (TNBC), are another crucial factor in therapeutic difficulties. While PARP inhibitors have demonstrated significant effectiveness in patients with germline BRCA mutations by taking advantage of homologous recombination repair deficiencies, BRCA2 mutations have been linked to lower overall and progression-free survival when treated with endocrine therapy ± CDK4/6 inhibitors, indicating different resistance profiles [184]. Furthermore, replication fork stability, homologous recombination repair, and decreased drug trapping can all lead to resistance to PARP inhibitors, which would limit long-term benefits [185]. BRCA mutant status adds complexity to TNBC, which is already marked by aggressive biology and therapeutic refractoriness, since there are still few viable therapy choices despite initial chemosensitivity. Figure 4 and Figure 5 present a schematic representation of BRCA1 and BRCA2 genes, while Figure 5B reveals what happens when there is a loss of a second BRCA allele in a BRCA mutation carrier.

#### 4.20.4. Genetic and Epigenetic Alterations

Somatic mutations in important oncogenes and tumor suppressor genes change therapeutic target and downstream signaling at the genomic level, allowing tumor cells to evade treatment pressure. The effectiveness of selective estrogen receptor modulators and degraders is compromised in hormone receptor-positive breast cancer due to constitutive ligand-independent signaling caused by activating mutations in the estrogen receptor gene (ESR1). After extended aromatase inhibitor treatment, ESR1 mutations are more common in metastatic cancers and are linked to poor outcomes because of ongoing proliferative signals [187]. Similarly, PIK3CA mutations or activation of the PI3K/AKT/mTOR pathway enable bypass signaling that maintains proliferation in the face of endocrine inhibition, resulting in both acquired and intrinsic resistance [188]. Targeted therapies are also challenged by genomic changes. Secondary mutations in the ERBB2 gene or downstream effectors can reduce sensitivity to tyrosine kinase inhibitors and monoclonal antibodies in HER2-positive malignancies, resulting in treatment failure. Similar to this, reversion mutations that restore homologous recombination repair, replication fork protection, or increased drug efflux led to resistance to PARP inhibitors in BRCA-associated and other homologous recombination-deficient cancers, thereby eliminating synthetic lethality [28,189].

In addition, adaptive resistance is supported by epigenetic dysregulation, which includes DNA methylation, histone modification, and chromatin remodelling, which modifies gene expression patterns without altering the genetic code. Drug sensitivity may be decreased by epigenetically silencing tumor suppressor genes and genes involved in drug absorption and apoptosis and activating survival pathways. For example, endocrine responsiveness is dampened by hypermethylation of estrogen-responsive regions and related transcriptional repressors, but stem-like phenotypes associated with broad treatment tolerance may be promoted by histone modifications [190].

#### 4.20.5. Hormone Receptor Mutations

Treatment challenges are also driven by hormone receptor abnormalities beyond ESR1 mutations. Alterations in progesterone receptor signaling and extensive crosstalk between estrogen receptor pathways and growth factor receptors such as HER2, insulin-like growth factor 1 (IGF-1), and fibroblast growth factor receptors (FGFR) can activate downstream signaling cascades including PI3K/AKT/mTOR and MAPK, thereby promoting resistance to endocrine and targeted therapies across multiple breast cancer subtypes [178,191]. These compensatory signaling networks frequently emerge during treatment and enable tumor cells to bypass therapeutic inhibition, highlighting the need for combination strategies capable of simultaneously targeting multiple resistance mechanisms [179]. Furthermore, epigenetic reprogramming, enhanced DNA damage response pathways, chromatin remodelling, and genomic alterations affecting therapeutic targets such as ERBB2 (HER2) contribute to the remarkable adaptability of breast cancer cells and support survival under sustained therapeutic pressure [179,180]. Tumor microenvironmental factors, including immune evasion, cancer-associated fibroblasts, extracellular matrix remodeling, and stromal–tumor interactions, further exacerbate therapeutic resistance by protecting resistant cellular subpopulations, promoting tumor plasticity, and facilitating disease recurrence and metastasis [192,193].

#### 4.20.6. Tumor Microenvironment

The tumor microenvironment (TME) plays a central role in the development and maintenance of therapeutic resistance in breast cancer. Composed of stromal cells, immune infiltrates, extracellular matrix components, and a complex network of secreted cytokines and growth factors, the TME creates a protective niche that shields tumor cells from immune surveillance and limits drug penetration [194,195]. Among the key cellular components of the TME, cancer-associated fibroblasts (CAFs) and tumor-associated macrophages (TAMs) promote resistance through the secretion of immunosuppressive cytokines, extracellular matrix remodelling, and activation of pro-survival signaling pathways that enhance tumor growth and diminish treatment efficacy [196,197]. Hypoxic regions within the TME further contribute to resistance by inducing adaptive transcriptional programs that promote angiogenesis, metabolic reprogramming, drug efflux, and the expression of survival-associated genes, thereby reducing intracellular drug accumulation and therapeutic effectiveness [194,195]. Moreover, the immunosuppressive nature of the TME compromises the efficacy of immunotherapies. Regulatory T cells and myeloid-derived suppressor cells suppress antitumor immune responses, inhibit cytotoxic T-cell activity, and facilitate immune escape, ultimately promoting tumor progression and therapeutic failure [198,199,200]. Collectively, these microenvironmental interactions foster the survival of resistant tumor cell populations, facilitate disease recurrence, and represent major obstacles to durable therapeutic responses in breast cancer [194,195,196]. This results in primary or secondary resistance to programmed death-ligand 1 (PD-1)/PD-L1 inhibitors in subsets of TNBC and other subtypes, as shown in Table 7.

### 4.21. Experimental Work Exploring Alternative Treatments

#### 4.21.1. Traditional Medicine in Breast Cancer Treatment

Herbal remedies derived from diverse plant sources contain a wide range of bioactive compounds that exhibit anticancer, anti-inflammatory, antioxidant, immunomodulatory, and pro-apoptotic activities, making them attractive candidates for cancer prevention and treatment [208,209]. In breast cancer research, numerous phytochemicals and herbal extracts have demonstrated the ability to suppress tumor cell proliferation, metastasis, angiogenesis, cancer stemness, and therapeutic resistance through the modulation of multiple molecular pathways [209,210]. Several natural products have also shown synergistic effects when combined with conventional chemotherapy, endocrine therapy, and targeted therapies, enhancing therapeutic efficacy while reducing treatment-associated toxicity and adverse effects [210,211]. Additionally, herbal formulations have been investigated as supportive therapies to improve immune function and mitigate chemotherapy- and radiotherapy-induced toxicities, including fatigue, nausea, myelosuppression, neuropathy, and dermatological complications, thereby improving patient quality of life [208,209].

Among the most extensively studied phytochemicals, epigallocatechin-3-gallate (EGCG), the principal catechin found in green tea, has demonstrated significant antitumor activity against breast cancer cells in both in vitro and in vivo models. EGCG induces apoptosis, inhibits cell-cycle progression, suppresses proliferation, and modulates the expression of key apoptotic regulators. In MCF-7 breast cancer cells, EGCG was shown to induce apoptosis and inhibit the G2/M cell-cycle transition through the suppression of miR-25, while restoration of miR-25 reversed these effects, confirming its involvement in EGCG-mediated anticancer activity [212]. Furthermore, in vivo studies demonstrated that EGCG significantly reduced tumor growth and enhanced apoptotic signaling, evidenced by decreased Ki-67 expression and increased poly(ADP-ribose) polymerase-1 (PARP-1) activity [212].More recent reviews continue to highlight EGCG as a promising natural compound for overcoming breast cancer progression and treatment resistance through its multitargeted effects on oxidative stress, inflammation, apoptosis, and oncogenic signaling pathways [210,213]. Beyond its direct anticancer effects, EGCG has also demonstrated clinical utility in supportive cancer care. A randomized clinical trial by Zhao et al. (2022) reported that prophylactic topical EGCG significantly reduced the incidence and severity of radiation-induced dermatitis in patients with breast cancer receiving adjuvant radiotherapy, suggesting an additional role for EGCG in reducing treatment-related toxicity and improving patient outcomes [214].

*Withaferin A* (WA), a potent steroidal lactone isolated from *Withania somnifera* (Ashwagandha), has emerged as a promising natural anticancer agent due to its ability to target multiple hallmarks of cancer. In breast cancer xenograft and MMTV-neu mouse models, WA significantly reduced tumor growth through activation of ERK/ribosomal S6 kinase signaling, upregulation of death receptor 5 (DR5), and enhanced nuclear localization of ETS-like transcription factor 1 (ELK1) and C/EBP homologous protein (CHOP), resulting in increased apoptotic cell death [215]. More recent studies have further highlighted the ability of WA to modulate key oncogenic pathways, including NF-κB, PI3K/AKT, Signal transducer and activator of transcription 3 (STAT3), Heat Shock Protein 90 (HSP90), and p53 signaling, thereby suppressing proliferation, metastasis, cancer stemness, and therapeutic resistance in breast cancer and other malignancies [216,217].

Similarly, baicalin and baicalein, two major flavonoids derived from *Scutellaria baicalensis,* have attracted considerable interest as potential therapeutic agents for breast cancer. Recent systematic reviews indicate that these compounds exert anticancer effects through the induction of apoptosis, inhibition of cell-cycle progression, suppression of metastasis, and modulation of signaling pathways such as PI3K/AKT/mTOR, NF-κB, and Wnt/β-catenin. In addition, they have demonstrated synergistic effects with conventional chemotherapeutic agents and show promise in nanoparticle-based delivery systems designed to improve bioavailability and therapeutic efficacy [218,219].

Baicalin has been shown to suppress metastasis in highly aggressive MDA-MB-231 breast cancer cells by targeting β-catenin signaling and reversing epithelial–mesenchymal transition (EMT), a critical process involved in cancer invasion and dissemination [218,220]. Furthermore, combined treatment with baicalin and baicalein enhanced apoptosis in MCF-7 cells through activation of caspase-9 and caspase-3, downregulation of B-cell lymphoma 2 (BCL-2), and upregulation of BAX and p53 via the ERK/p38 MAPK signaling pathway [218]. Recent mechanistic analyses have further identified molecular targets associated with baicalein-mediated breast cancer inhibition, including heat shock protein 90 alpha family class A member 1 (HSP90AA1), cyclin B1 (CCNB1), and nuclear receptor coactivator 2 (NCOA2), supporting its role as a multitarget therapeutic agent [218,221].

A study by Chung and colleagues revealed that *Oldenlandia diffusa* possesses antimetastatic properties by reducing the invasive capacity of MCF-7 breast cancer cells through inhibition of phosphorylated extracellular signal-regulated kinase (p-ERK), p38, and nuclear factor-κB (NF-κB) signaling pathways, accompanied by downregulation of matrix metalloproteinase-9 (MMP-9) and intercellular adhesion molecule 1 (ICAM-1) expression and induction of apoptosis [222]. More recent reviews have confirmed that *O. diffusa* exerts broad anticancer activities through modulation of inflammatory signaling, oxidative stress, apoptosis, metastasis-related pathways, and tumor–immune interactions, supporting its potential role in breast cancer management [221,223].

Additionally, Ginsenoside Rh2, a prominent bioactive constituent of red ginseng, has been reported to inhibit the proliferation of MCF-7 breast cancer cells in a dose-dependent manner through epigenetic regulation of genes associated with tumorigenesis, including the upregulation of ST3 beta-galactoside alpha-2,3-sialyltransferase 4 (ST3GAL4)ST3GAL4, C1orf198, and CLINT1 [34]. Recent studies have further demonstrated that Rh2 suppresses cancer cell growth by regulating apoptosis, autophagy, cancer stemness, and immune-related signaling pathways [224,225].

Similarly, ginsenoside Rg3, an active compound generated during the heat processing of ginseng, significantly inhibited the proliferation of MDA-MB-231 and MCF-7 breast cancer cells by decreasing the expression of cyclin D1 and cyclin A and inducing G1-phase cell-cycle arrest [226]. Recent evidence has shown that Rg3 exerts multifaceted anticancer effects through the regulation of apoptosis, angiogenesis, immune responses, epithelial–mesenchymal transition, cancer stem cell maintenance, and PI3K/AKT- and NF-κB-related signaling pathways, thereby suppressing tumor progression and therapeutic resistance [227,228]. In breast cancer specifically, Rg3 has also been reported to reduce the stemness of breast cancer stem cells through activation of the Hippo signaling pathway, highlighting its potential as a therapeutic adjuvant [229].

In other studies, garlic (*Allium sativum*) and its organosulfur derivatives have been widely reported to inhibit the growth of breast cancer cells through the induction of apoptosis, cell-cycle arrest, autophagy, and modulation of multiple oncogenic signaling pathways. Recent evidence highlights compounds such as allicin, diallyl sulfide, diallyl disulfide, and diallyl trisulfide (DATS) as potent multitarget agents capable of suppressing proliferation, angiogenesis, metastasis, and therapeutic resistance in breast cancer models [230,231,232]. Diallyl trisulfide, in particular, has demonstrated significant antiproliferative activity through activation of apoptotic pathways involving p53, BAX, and caspase signaling, supporting its potential application in breast cancer therapy [209,230].

Curcumin, a bioactive polyphenol derived from *Curcuma longa*, exhibits broad-spectrum anticancer activity by suppressing breast cancer cell proliferation, invasion, metastasis, angiogenesis, and cancer stemness. Recent reviews have demonstrated that curcumin exerts these effects through the regulation of NF-κB, PI3K/AKT/mTOR, JAK/STAT, Wnt/β-catenin, and MAPK signaling pathways while also enhancing the efficacy of conventional chemotherapy and reducing drug resistance [178,233]. Furthermore, curcumin-based nanoformulations have shown improved bioavailability and therapeutic efficacy in preclinical breast cancer models, highlighting their translational potential [233].

Beyond Asian herbal products, African medicinal plants have shown considerable promise in experimental breast cancer models. Multiple studies on *Calliandra portoricensis* have demonstrated significant antiproliferative and anti-mammary tumor activities mediated through pro-apoptotic, anti-inflammatory, and antioxidant mechanisms [234,235]. Fractionated and solvent-specific extracts of *C. portoricensis* markedly attenuated mammary tumor progression by suppressing inflammatory responses and enhancing apoptosis-related signaling pathways *in vitro* and *in vivo* [235].

Similarly, phytochemical investigations of *Annona muricata* (soursop) continue to support its anticancer potential. Recent systematic reviews and experimental studies have shown that *A. muricata* extracts and acetogenins possess antiproliferative, pro-apoptotic, antioxidant, and antimetastatic properties against multiple cancer types, including breast cancer, through modulation of mitochondrial function, oxidative stress, and cell survival pathways [236,237]. These findings support the continued investigation of *A. muricata* as a source of novel therapeutic agents for breast cancer management.

#### 4.21.2. Gene Therapy Strategies for Breast Cancer Treatment

Cancer remains one of the leading causes of morbidity and mortality worldwide and continues to pose a major public health challenge despite substantial advances in prevention, diagnosis, and treatment [15,238]. Extensive research has explored the molecular mechanisms underlying cancer development and progression, leading to the emergence of numerous therapeutic strategies. However, the global cancer burden continues to increase due to population growth, aging, environmental exposures, and lifestyle-related risk factors [15]. Cancer arises from a disruption of normal cellular homeostasis characterized by uncontrolled cell proliferation, evasion of programmed cell death, impaired differentiation, sustained angiogenesis, and enhanced metastatic potential. These hallmarks are driven by genetic and epigenetic alterations affecting oncogenes, tumor suppressor genes, DNA repair genes, and signaling pathways that regulate cellular growth and survival [239]. Among the most extensively studied tumor suppressor genes are TP53 and PTEN (phosphatase and tensin homolog), which play crucial roles in maintaining genomic stability, regulating cell-cycle progression, promoting apoptosis, and suppressing tumor development. Loss or mutation of these genes is frequently associated with cancer initiation, progression, treatment resistance, and poor clinical outcomes, making them important targets for cancer therapeutics and gene-based treatment strategies [240].

Initially, monogenic genetic disorders were the primary focus of gene therapy; however, over time, efforts have expanded toward the treatment of cancer, where solid and hematological malignancies now account for a major proportion of clinical gene therapy trials [241]. Because genetic alterations and gene expression dysregulation play central roles in breast cancer initiation and progression, gene-based therapeutic strategies have shown increasing promise in this disease context [242].

Gene therapy approaches are generally classified into several major categories as follows:

- Mutation Compensation/Gene Replacement: Tumor suppressor genes such as TP53, RB, and BRCA1/2 can be restored through functional gene delivery to re-establish normal cell-cycle control and promote apoptosis in cancer cells [243,244].
- Oncolytic Virotherapy: This strategy involves the use of genetically engineered viruses (e.g., adenovirus, herpes simplex virus) that selectively infect and lyse tumor cells while sparing normal tissues, while also stimulating antitumor immune responses [245,246].
- Suicide Gene Therapy/Molecular Chemotherapy: This approach introduces genes encoding enzymes such as cytosine deaminase (CD), herpes simplex virus thymidine kinase (HSV-TK), cytochrome P450-2B1, and nitroreductase, which convert non-toxic prodrugs into cytotoxic agents specifically within tumor tissues, thereby enhancing targeted tumor killing and limiting systemic toxicity [247].
- Genetic Immunopotentiation: This involves enhancing antitumor immunity through delivery of cytokine genes (e.g., IL-12, GM-CSF) or through adoptive cell therapies such as chimeric antigen receptor (CAR) T-cell therapy targeting breast cancer-associated antigens such as HER2 and MUC1, improving immune-mediated tumor clearance [248,249].
- Gene Silencing and Editing: Advanced technologies such as siRNA/shRNA-mediated gene knockdown and CRISPR/Cas9 genome editing are being used to suppress oncogenes (e.g., c-MYC) or permanently correct cancer-associated mutations, offering precise and potentially curative approaches to breast cancer therapy [250,251].

Lately, gene therapy strategies have entered early-phase clinical trials for a variety of diseases, including breast cancer, where they are being explored as either direct anti-tumor approaches or immune-modulating strategies, as shown in Table 8. Recent studies and ongoing Phase I/II trials demonstrate increasing use of viral vector–based gene delivery systems and cytokine-encoding constructs, particularly in combination with immunotherapy and chemotherapy to enhance anti-tumor immune responses [252,253,254,255].

#### 4.21.3. Next-Generation Sequencing Breast Cancer Therapy

The advent of next-generation sequencing (NGS) technologies in cancer research has significantly advanced the understanding of neoplasia by enabling comprehensive genomic, transcriptomic, and epigenomic profiling of oncogenes, tumor suppressor genes, and mutational processes across diverse cancers, including breast cancer [256,257]. These technologies include chromatin immunoprecipitation sequencing (ChIP-seq), reduced representation bisulfite sequencing (RRBS), RNA sequencing (RNA-seq), targeted exome sequencing, hotspot sequencing, whole genome sequencing (WGS), and whole exome sequencing (WES), all of which collectively provide high-resolution insights into cancer heterogeneity and evolution [258,259]. In breast cancer, large-scale NGS studies have predominantly focused on invasive breast carcinoma of no special type (IBC-NST), invasive lobular carcinoma (ILC), and mixed ductal–lobular carcinoma, revealing subtype-specific mutational landscapes, clonal evolution patterns, and therapeutic resistance mechanisms [260,261].

Precision medicine in breast cancer involves customising treatment to patients based on genetics, tumor characteristics, and other variables. Clinicians can use modern technology, such as genomic sequencing, to uncover genetic abnormalities and guide therapy [262]. These methods improve results by enabling the selection of personalized medicines with fewer adverse effects. For instance, Kawaji and colleagues utilized NGS to examine the genomic profiles of 115 advanced and metastatic breast cancer tissue samples. This analysis permits the identification of actionable modifications, like short variants in TP53, PIK3CA, GATA3, PTEN, and structural variants in ERBB2, MYC, RAD21, and CCND1. They further evaluated whether the variants indicated the use of drugs based on medical evidence. The results allowed the identification of patients who will benefit from poly ADP-ribose polymerase (PARP) inhibitors and DNA-damaging medications, as well as those with BRCA1/2 function-suppressing mutations [263].

Furthermore, a study by Luen and colleagues involving targeted NGS in a large cohort of 1276 pre-menopausal HR+/HER2− patients highlighted that younger patients (<40  years, *n*  =  359) harboured low levels of mutations in *PIK3CA* (32% versus 47%), *CDH1* (3% versus 9%), and *MAP3K1* (7% versus 12%), and elevated mutation rates in *GATA3* (19% versus 16%) and *TP53* (7% versus 3%, *q*  <  0.010). Copy Number Alterations (CNAs) were also higher than in their older counterparts (47% versus 26%). *PIK3CA* mutations, an oncogenic driver gene that vary substantially in prognosis between women under 40 and those over 40 years (HR 1.78, 95% CI 1.08–2.92), with *p*  <  0.002 [264,265]. Moreover, a group of researchers employed whole-exome sequencing on a cohort of 187 young Korean breast cancer patients (88.2% were pre-menopausal), and the findings were compared with primarily Caucasian and post-menopausal breast cancer cohort (TCGA) whole-exome sequencing data (72.3% were post-menopausal). The younger patients had increased enrichment of somatic changes in tumor protein p53 (47.9% vs. 32%), phosphatidylinositol-4,5-bisphosphate 3-kinase, catalytic subunit alpha (28.5% vs. 32%), and GATA binding protein 3 (12.4% vs. 9.1%). In addition, there was an elevated prevalence of BRCA1/2 germline mutations in younger patients, which affected 13.7% of young patients (age ≤ 40 years) but only 4.8% of the intermediate age group (age > 40 years) (*p*  =  0.08) and 3.4% of the TCGA cohort of the older age group (age > 60 years) (*p*  =  6.85 × 10^−5^) [266].

Several triple-negative breast cancer (TNBC) diagnosis stratifications have been investigated and identified using NGS techniques for biomarkers. Furthermore, [267] conducted an NGS survey utilising the Illumina platform on the plasma TNBC patients without special type (NST) and special type (ST). In this study, 89 TNBC patients (72 NSTs and 17 STs) had the mutation statuses of 520 cancer-related genes. The findings from this study revealed that TP53 (88.7%), PIK3CA (26.8%), and MYC (18.3%) were the most commonly mutated genes in NST, whereas TP53 (68.8%), PIK3CA (50%), JAK3 (18.8%), and KMT2C (18.8%) were the most frequently mutated genes in ST, according to the sequencing data. The most frequently mutated genes in both groupings are TP53 and PIK3CA, while ST has far lower TP53 and higher PIK3CA mutation rates than NST. This discovery offered genetic proof that the tumor growth in these two TNBC subgroups is caused by somewhat distinct molecular processes. Furthermore, STs may benefit more from medications that target the PIK3CA pathway than NSTs [267].

An intriguing study by Dillon and colleagues involved an NGS assay called JAX-CTP to determine the mutation patterns of 20 TNBC patients. A clinically validated panel of SNPs, copy number variants, insertions, and deletions often seen in 358 cancer-related genes served as the basis for this assay [268]. Furthermore, MYC amplification was found in 75% of the patients examined by Illumina sequencing of formalin-fixed, paraffin-embedded (FFPE) tissues, whereas TP53, Aurora kinase A (AURKA), and Kinase Insert Domain Receptor (KDR) mutations were found in 6 out of 20 cases (30%) in a previous study [268]. About 15% of all human genes, including those involved in cell survival and proliferation, are regulated by the transcription factor MYC. In fact, MYC dysregulation contributes to angiogenesis, tumor cell immortalisation, and the epithelial–mesenchymal transition (EMT) in the basal-like immune-suppressed (BLIS) and basal-like immune-activated (BLIA) subsets of TNBC [265,269]. Thus, effective communication and positive cooperation among all stakeholders are critical to the success of NGS and precision oncology [270].

## 5. Discussion

### Critical Perspectives, Current Controversies, and Future Challenges in Breast Cancer Diagnosis and Treatment

Over the past 20 years, there has been a significant shift in the management of breast cancer, moving from morphology-based classification and widely used treatments to molecularly guided therapeutic approaches. Despite these developments, several disputes and unsolved issues still prevent scientific advancements from consistently improving patient outcomes. Molecular categorization systems, treatment resistance, precision oncology implementation, and the clinical adoption of new therapeutic technologies are areas where these difficulties are more noticeable. The molecular classification of breast cancer into luminal A, luminal B, HER2-enriched, and triple-negative subtypes has transformed clinical decision-making by enabling biologically informed therapeutic strategies tailored to tumor subtype [3,60,61,157].

However, these categories do not fully capture the substantial intertumoral and intratumoral heterogeneity that characterizes breast cancer. Although molecular subtypes provide important prognostic and predictive information, individual tumors often exhibit considerable genomic, transcriptomic, and clonal diversity, resulting in heterogeneous treatment responses among patients classified within the same subtype [60,61,271].

A major point of contention is the inconsistency between immunohistochemical and genomic classification schemes. For example, triple-negative breast cancer (TNBC), once considered a single clinical entity, is now recognized as a heterogeneous disease comprising multiple molecular and transcriptomic subtypes with distinct biological behaviours and therapeutic vulnerabilities [60,61,272,273]. Although TNBC subtyping frameworks, such as those proposed by Lehmann and colleagues [274], have improved biological understanding of the disease, they have not been widely adopted in routine clinical practice due to limited reproducibility, lack of standardized implementation, and the absence of fully validated subtype-specific therapeutic strategies [60,275,276]. Similarly, the recognition of HER2-low breast cancer has challenged the traditional binary classification of HER2-positive and HER2-negative disease. The demonstrated clinical efficacy of antibody–drug conjugates, particularly trastuzumab deruxtecan, in patients with HER2-low tumors highlights that clinically meaningful responses can occur even in the absence of HER2 overexpression or gene amplification [61,98].

These findings suggest that conventional HER2 classification may no longer fully capture therapeutic opportunities and highlight the need for more refined biomarker-driven approaches to breast cancer classification [60,98,277]. As a result, future breast cancer classification systems are likely to move beyond static receptor-based criteria by integrating dynamic genomic, transcriptomic, proteomic, and tumor microenvironmental data.

Advances in next-generation sequencing (NGS), liquid biopsy technologies, and multigene expression assays have facilitated improved risk stratification and more personalized treatment strategies. However, substantial barriers remain before precision oncology can be widely implemented in routine clinical practice. Although assays such as Oncotype DX, MammaPrint, and NGS-based profiling can identify recurrence risk and actionable genomic alterations, their clinical adoption remains uneven, particularly in low- and middle-income settings, due to high costs, limited access to sequencing technologies, reimbursement constraints, and insufficient bioinformatics infrastructure [60,278].

Consequently, the successful integration of precision oncology into routine breast cancer management will depend not only on continued technological advances but also on improving affordability, standardisation, equitable access, and the clinical interpretation of complex molecular data.

Another major challenge in precision oncology is the clinical interpretation of increasingly complex genomic datasets. Although next-generation sequencing (NGS) frequently identifies potentially actionable genomic alterations, many detected variants remain of uncertain clinical significance [271,278], limiting their immediate therapeutic utility. In addition, the molecular landscape of breast cancer evolves over time through clonal evolution and treatment-induced selective pressures [60,279,280], meaning that actionable alterations identified in the primary tumor may differ from those present in recurrent or metastatic disease. This temporal and spatial heterogeneity raises important questions regarding the optimal timing and frequency of molecular testing throughout disease progression. Liquid biopsy technologies offer a promising, minimally invasive approach for longitudinal disease monitoring and the detection of emerging resistance mechanisms; however, challenges related to analytical standardisation, assay sensitivity in early-stage disease, clinical validation, and cost-effectiveness continue to limit their widespread clinical implementation [44,278,281].

Consequently, despite its limitations in capturing tumor heterogeneity, tissue biopsy remains the diagnostic gold standard. Furthermore, although targeted therapies, endocrine therapies, PARP inhibitors, immunotherapy, and antibody–drug conjugates have substantially improved clinical outcomes, both intrinsic and acquired resistance remain major barriers to durable therapeutic benefit. Treatment resistance is increasingly recognized as a multifactorial process involving genomic alterations, epigenetic reprogramming, activation of compensatory signalling pathways, pharmacogenomic variability, tumor plasticity, and dynamic interactions within the tumor microenvironment [60,61,271,282,283].

In hormone receptor–positive breast cancer, resistance to endocrine therapy commonly arises through activating ESR1 mutations and crosstalk between oestrogen receptor signalling and growth factor pathways, resulting in disease progression despite continued oestrogen suppression [282,284,285]. Similarly, resistance to HER2-targeted therapies develops through multiple mechanisms, including activation of downstream signalling pathways, alterations in HER2, and adaptive signalling network rewiring [61,233]. Even therapies based on synthetic lethality, such as PARP inhibitors, ultimately encounter acquired resistance through restoration of homologous recombination repair, replication fork protection, and other compensatory DNA damage response mechanisms [145,286]. In triple-negative breast cancer (TNBC), marked genomic instability, intratumoral heterogeneity, and aggressive tumor biology contribute to rapid therapeutic escape despite initial sensitivity to chemotherapy [276,287]. Collectively, these diverse resistance mechanisms highlight the limitations of targeting a single molecular pathway and support an increasing shift towards biomarker-guided combination therapies designed to inhibit complementary signalling pathways, exploit tumor vulnerabilities, and delay or prevent the emergence of therapeutic resistance [60,61].

Emerging therapeutic strategies, including antibody–drug conjugates (ADCs), personalised cancer vaccines, mRNA-based therapies, tumor microenvironment (TME)-targeted approaches, nanomedicine, and gene-editing technologies, represent promising advances in breast cancer treatment [6,61,98]. Nevertheless, many of these approaches remain in the early stages of clinical translation, and their long-term efficacy, safety, and cost-effectiveness have yet to be fully established. ADCs have demonstrated substantial clinical benefit in HER2-positive, HER2-low, and triple-negative breast cancers; however, acquired resistance, optimal biomarker selection, toxicity management, and treatment sequencing remain important clinical challenges [61,98,288].

Although epigenetic therapies and tumor microenvironment (TME)-targeted strategies have shown encouraging preclinical and early clinical efficacy, their broader clinical adoption remains constrained by the lack of validated predictive biomarkers for patient selection [289,290]. Future advances in breast cancer diagnosis and treatment will rely on biomarker-driven clinical trials and integrated multi-omics approaches to improve patient stratification, optimise treatment selection, and overcome tumor heterogeneity and therapeutic resistance [60,291].

CRISPR-based gene-editing approaches face substantial barriers to clinical translation, including limitations in delivery efficiency, off-target effects, immunogenicity, and regulatory complexity [65,292]. Similarly, nanomedicine-based drug delivery systems are hindered by challenges related to manufacturing scalability, pharmacokinetic variability, reproducibility, regulatory standardisation, and clinical translation despite their promising preclinical efficacy [292,293].

Future success in breast cancer care will depend not only on static molecular classifications but also on adaptive models that integrate genomic, transcriptomic, epigenetic, immunological, and tumor microenvironmental data. It will be equally crucial to develop robust biomarkers capable of predicting therapeutic response, resistance emergence, and disease recurrence in a longitudinal and clinically actionable manner. The integration of liquid biopsy technologies, AI-assisted analytics, serial molecular monitoring, and multi-omics profiling may enable the development of dynamic, patient-specific treatment strategies [44,278,294]. However, scientific innovation alone will not eliminate global disparities in breast cancer outcomes. The implementation of molecular diagnostics, targeted therapies, and precision oncology approaches remains limited by cost, infrastructure, and workforce constraints, particularly in low- and middle-income countries [47,60].

## 6. Conclusions

Significant regional differences in incidence, death, and survival, as well as deep biological variability and changing molecular subtypes, make breast cancer a persistent worldwide health concern. Tumor classification, prognostication, and therapeutic decision-making have been significantly improved by developments in molecular genetics, biomarker discovery, and next-generation sequencing. This has made it possible to move away from conventional “one-size-fits-all” approaches and toward more accurate and individualized treatment plans. While emerging platforms like liquid biopsy, multi-omics integration, and pharmacogenomics provide real-time insights into tumor evolution and treatment response, targeted therapies, immunotherapies, antibody–drug conjugates, PARP inhibitors, and CDK4/6 inhibitors especially when used in rational combinations have significantly improved clinical outcomes in specific patient populations.

Despite this advancement, long-term survival gains are still constrained by therapeutic resistance, disease recurrence, aggressive subtypes like triple-negative breast cancer, and unequal access to cutting-edge diagnostics and treatments, particularly in low- and middle-income nations. These difficulties underscore the importance of combination tactics, targeting of the tumor microenvironment, epigenetic modification, and novel delivery methods, such as platforms based on nanotechnology. Simultaneously, the usefulness of complementary and economical treatments that may increase efficacy while lowering toxicity is shown by experimental evidence supporting medicinal plants, bioactive chemicals, drug repurposing, gene therapy, and new mRNA-based and vaccination approaches.

In general, integrating molecularly guided medicines with scalable diagnostic technologies, bolstering the capacity of the health system, and promoting interdisciplinary and international collaboration will be necessary for long-term improvements in breast cancer outcomes. To overcome resistance, reduce survival inequalities, and eventually improve both survival and quality of life for patients with breast cancer worldwide, more translational research, equitable precision medicine implementation, and context-specific innovation are crucial.

## Figures and Tables

**Table 1:** The age-standardised mortality-to-incidence ratio globally.

Country	ISO Code	ASR Mortality per 100,000 People	ASR Incidence per 100,000 People	MIR (Mortality/Incidence)
Afghanistan	4	17.90	29.40	0.609
Albania	8	14.50	51.10	0.284
Algeria	12	20.80	61.90	0.336
Angola	24	14.30	29.40	0.486
Azerbaijan	31	11.80	32.90	0.359
Argentina	32	17.60	71.30	0.247
Australia	36	12.30	101.50	0.121
Austria	40	14.30	69.50	0.206
Bahamas	44	31.70	64.60	0.491
Bahrain	48	19.70	58.50	0.337
Bangladesh	50	7.60	15.20	0.500
Armenia	51	16.10	39.60	0.407
Barbados	52	29.80	57.70	0.516
Belgium	56	14.20	104.40	0.136
Bhutan	64	2.30	4.60	0.500
Bolivia	68	7.50	26.80	0.280
Bosnia Herzegovina	70	15.00	51.90	0.289
Botswana	72	8.30	23.00	0.361
Brazil	76	13.90	63.10	0.220
Belize	84	11.70	46.80	0.250
Solomon Islands	90	20.40	49.20	0.415
Brunei Darussalam	96	14.30	50.20	0.285
Bulgaria	100	15.50	52.80	0.294
Myanmar	104	10.10	23.40	0.432
Burundi	108	15.70	25.30	0.621
Belarus	112	13.30	56.60	0.235
Cambodia	116	11.20	25.10	0.446
Cameroon	120	27.40	48.60	0.564
Canada	124	13.40	88.60	0.151
Cape Verde	132	7.80	23.40	0.333
Central African Republic	140	25.30	37.70	0.671
Sri Lanka	144	11.20	27.70	0.404
Chad	148	8.80	14.20	0.620
Chile	152	10.30	38.20	0.270
China	156	6.10	33.00	0.185
Colombia	170	13.30	50.70	0.262
Comoros	174	11.80	21.50	0.549
Congo, The Republic of Congo	178	12.50	26.90	0.465
The Democratic Republic of Congo	180	15.60	26.50	0.589
Costa Rica	188	10.60	37.50	0.283
Croatia	191	11.70	74.60	0.157
Cuba	192	13.90	47.20	0.294
Cyprus	196	18.60	104.80	0.177
Czechia	203	11.70	72.50	0.161
Benin	204	18.90	33.30	0.568
Denmark	208	14.10	95.40	0.148
Dominican Republic	214	23.00	53.40	0.431
Ecuador	218	11.20	39.50	0.284
El Salvador	222	7.80	39.70	0.196
Equatorial Guinea	226	21.40	41.00	0.522
Ethiopia	231	24.00	40.80	0.588
Eritrea	232	22.40	37.80	0.593
Estonia	233	13.20	63.10	0.209
Fiji	242	38.90	60.30	0.645
Finland	246	11.90	92.30	0.129
France (metropolitan)	250	15.80	105.40	0.150
French Guyana	254	15.90	55.70	0.285
French Polynesia	258	23.20	71.90	0.323
Djibouti	262	22.10	38.10	0.580
Gabon	266	13.20	36.40	0.363
Georgia	268	20.60	49.00	0.420
The Republic of the Gambia	270	6.60	12.10	0.545
Gaza Strip and West Bank	275	19.70	46.30	0.425
Germany	276	15.80	77.00	0.205
Ghana	288	19.30	40.20	0.480
Greece	300	14.70	82.40	0.178
France, Guadeloupe	312	16.70	66.40	0.252
Guam	316	17.00	44.20	0.385
Guatemala	320	6.80	28.40	0.239
Guinea	324	8.90	16.00	0.556
Guyana	328	15.20	52.60	0.289
Haiti	332	14.30	25.20	0.567
Honduras	340	11.20	27.30	0.410
Hungary	348	16.90	76.40	0.221
Iceland	352	17.10	71.10	0.241
India	356	13.70	26.60	0.515
Indonesia	360	14.40	41.80	0.344
Iran	364	11.00	30.50	0.361
Iraq	368	23.50	56.90	0.413
Ireland	372	17.20	91.50	0.188
Israel	376	16.20	78.70	0.206
Italy	380	14.80	87.00	0.170
Côte d’Ivoire	384	25.40	45.40	0.559
Jamaica	388	35.20	71.10	0.495
Japan	392	9.70	74.40	0.130
Kazakhstan	398	12.30	36.90	0.333
Jordan	400	19.30	60.00	0.322
Kenya	404	19.60	40.80	0.480
Korea, DPR	408	9.80	32.30	0.303
Korea, Republic of	410	5.80	61.50	0.094
Kuwait	414	17.00	49.80	0.341
Kyrgyzstan	417	8.20	25.30	0.324
Lao PDR	418	12.60	31.60	0.399
Lebanon	422	20.40	57.90	0.352
Lesotho	426	9.80	18.90	0.519
Latvia	428	16.30	66.00	0.247
Liberia	430	19.50	33.60	0.580
Libya	434	13.30	31.00	0.429
Lithuania	440	14.50	62.70	0.231
Luxembourg	442	15.10	99.70	0.151
Madagascar	450	16.40	30.30	0.541
Malawi	454	14.80	25.20	0.587
Malaysia	458	19.30	46.10	0.419
Maldives	462	13.20	39.80	0.332
Mali	466	23.10	37.70	0.613
Malta	470	13.80	86.30	0.160
France, Martinique	474	16.30	77.20	0.211
Mauritania	478	18.50	34.50	0.536
Mauritius	480	19.00	52.70	0.361
Mexico	484	10.30	39.90	0.258
Mongolia	496	3.20	12.60	0.254
Republic of Moldova	498	17.50	46.60	0.376
Montenegro	499	23.30	76.50	0.305
Morocco	504	18.10	58.40	0.310
Mozambique	508	12.00	19.70	0.609
Oman	512	13.10	34.00	0.385
Namibia	516	22.50	53.90	0.417
Nepal	524	7.60	14.40	0.528
The Netherlands	528	14.50	101.60	0.143
New Caledonia	540	16.80	90.20	0.186
Vanuatu	548	8.90	29.60	0.301
New Zealand	554	15.50	94.40	0.164
Nicaragua	558	10.60	35.30	0.300
Niger	562	19.00	29.50	0.644
Nigeria	566	26.80	51.50	0.520
Norway	578	11.00	95.60	0.115
Pakistan	586	18.60	34.20	0.544
Panama	591	10.50	41.40	0.254
Papua New Guinea	598	26.70	46.00	0.580
Paraguay	600	16.90	58.40	0.289
Peru	604	9.40	39.30	0.239
Philippines	608	21.50	60.30	0.357
Poland	616	17.40	66.00	0.264
Portugal	620	14.50	88.80	0.163
Guinea-Bissau	624	16.70	27.20	0.614
Timor-Leste	626	10.20	26.40	0.386
Puerto Rico	630	14.30	62.30	0.230
Qatar	634	11.40	39.00	0.292
France, La Réunion	638	12.10	55.90	0.216
Romania	642	16.50	69.20	0.238
Russian Federation	643	13.60	57.70	0.236
Rwanda	646	8.40	16.20	0.519
Saint Lucia	662	16.40	51.70	0.317
Sao Tome and Principe	678	9.60	19.90	0.482
Saudi Arabia	682	7.60	25.30	0.300
Senegal	686	16.40	29.90	0.548
Serbia	688	18.50	60.50	0.306
Sierra Leone	694	4.20	7.00	0.600
Singapore	702	17.80	72.60	0.245
Slovakia	703	18.10	67.30	0.269
Viet Nam	704	14.70	38.00	0.387
Slovenia	705	15.00	82.30	0.182
Somalia	706	25.70	38.60	0.666
South Africa	710	17.00	47.80	0.356
Zimbabwe	716	18.60	35.80	0.520
Spain	724	10.60	81.00	0.131
South Sudan	728	17.60	28.00	0.629
Sudan	729	20.70	39.90	0.519
Suriname	740	14.40	46.40	0.310
Eswatini	748	11.30	26.20	0.431
Sweden	752	11.90	81.40	0.146
Switzerland	756	12.40	77.40	0.160
Syrian Arab Republic	760	20.90	46.20	0.452
Tajikistan	762	7.40	19.50	0.379
Thailand	764	11.80	37.40	0.316
Togo	768	18.10	32.90	0.550
Trinidad and Tobago	780	22.10	54.00	0.409
United Arab Emirates	784	15.90	57.10	0.278
Tunisia	788	10.90	36.20	0.301
Türkiye	792	12.50	46.80	0.267
Turkmenistan	795	15.60	31.10	0.502
Uganda	800	12.60	23.30	0.541
Ukraine	804	13.20	43.10	0.306
North Macedonia	807	19.50	65.30	0.299
Egypt	818	19.90	55.40	0.359
United Kingdom	826	14.00	94.00	0.149
Tanzania	834	12.90	25.10	0.514
United States of America	840	12.20	95.90	0.127
Burkina Faso	854	13.20	21.40	0.617
Uruguay	858	21.60	75.10	0.288
Uzbekistan	860	13.00	27.80	0.468
Venezuela	862	16.20	47.10	0.344
Samoa	882	28.10	88.80	0.316
Yemen	887	14.00	25.40	0.551
Zambia	894	10.80	22.30	0.484

**Table 2:** Major molecular subtypes of breast cancer, key biomarkers and approximate prognosis and approved targeted therapies.

Molecular Subtype	Key Biomarkers	Approximate Prognosis	Approved Targeted Therapies
Luminal A	ER+, PR high, HER2−, low Ki-67	Best prognosis; low recurrence risk; endocrine-sensitive [31,32]	Endocrine therapy (tamoxifen, aromatase inhibitors, fulvestrant), cyclin-dependent kinase (CDK)4/6 inhibitors in advanced disease [31,33]
Luminal B (HER2−)	ER+, PR low/negative, HER2−, high Ki-67	Intermediate prognosis; higher recurrence risk than Luminal A [31,32]	Endocrine therapy + CDK4/6 inhibitors (palbociclib, ribociclib, abemaciclib); PI3K inhibitor (alpelisib for PIK3CA-mutant tumors); AKT inhibitor (capivasertib) [31,33]
Luminal B (HER2+)/Triple-positive	ER+, PR±, HER2+	Worse than Luminal A; better than untreated HER2+ disease due to effective targeted therapy [32,33]	Anti-HER2 therapy (trastuzumab, pertuzumab, trastuzumab deruxtecan, tucatinib) combined with endocrine therapy and/or chemotherapy [31,33]
HER2-enriched	ER−, PR−, HER2+	Historically poor prognosis; markedly improved with HER2-targeted therapy [31,32]	Trastuzumab, pertuzumab, trastuzumab emtansine (T-DM1), trastuzumab deruxtecan (T-DXd), tucatinib, lapatinib, neratinib [33,34]
Triple-negative/Basal-like (TNBC)	ER−, PR−, HER2−	Generally poorest prognosis; high recurrence risk within first 3–5 years [35,36,37]	Immune checkpoint inhibitors (pembrolizumab for eligible patients), PARP inhibitors (olaparib, talazoparib for germline BRCA1/2 mutations), antibody–drug conjugates (sacituzumab govitecan) [36,37].

**Table 3:** Comparison of the key characteristics of hereditary, familial, and sporadic breast cancer.

Characteristics	Hereditary Breast Cancer	Familial Breast Cancer (Familial Clustering)	Sporadic Breast Cancer
Frequency	~5–10% of breast cancers	~15–25% of breast cancers	~70–80% of breast cancers
Genetic basis	Germline pathogenic variant in a high-penetrance susceptibility gene	No identifiable high-penetrance mutation; likely polygenic inheritance and/or shared environmental factors	No inherited predisposition; caused by acquired somatic mutations
Family history	Strong family history, often involving multiple generations	More cases in a family than expected by chance, but inheritance pattern unclear	Usually absent or not significant
Inheritance pattern	Often autosomal dominant with incomplete penetrance	No clear Mendelian pattern	Not inherited
Age at onset	Often younger (<50 years)	Intermediate; may be somewhat younger than population average	Usually older age
Associated cancers	May include ovarian, pancreatic, prostate, melanoma, etc., depending on the gene involved	Generally limited to breast cancer clustering	Usually isolated breast cancer
Examples of genes	BRCA1, BRCA2, PALB2, TP53, PTEN, CDH1, STK11	Multiple low-risk alleles identified through GWAS; no single causative mutation	Somatic mutations acquired during life (e.g., PIK3CA, TP53, ESR1)
Genetic testing	Frequently reveals a pathogenic germline variant	Usually negative for known high-risk genes	Usually negative for inherited susceptibility genes

**Table 4:** (**A**) Hereditary susceptibility breast cancer genes used as biomarkers for disease. (**B**) Predictive and prognostic breast cancer genes used as biomarkers for disease.

(**A**)			
**Gene Symbol**	**Full Name**	**Biomarker Type**	**Clinical Significance**
ATM	Ataxia Telangiectasia Mutated	Susceptibility (Moderate)	DNA damage repair, moderate risk of familial cancer
BRCA1	Breast Cancer Gene 1	Susceptibility (High Risk)	Hereditary cancer risk, PARP inhibitor response
BRCA2	Breast Cancer Gene 2	Susceptibility (High Risk)	Hereditary cancer risk, PARP inhibitor response
CDH1	E-Cadherin 1	Susceptibility (High)	Hereditary diffuse gastric cancer/lobular breast cancer
CHEK2	Checkpoint Kinase 2	Susceptibility (Moderate)	Cell cycle regulation, familial cancer risk
PALB2	Partner and Localizer of BRCA2	Susceptibility (Moderate/High)	Hereditary risk, DNA repair dysfunction
(**B**)			
**Gene Symbol**	**Full Name**	**Biomarker Type**	**Clinical Significance**
ERBB2 (HER2)	Erb-B2 Receptor Tyrosine Kinase 2	Predictive/Prognostic	HER2-targeted therapy (e.g., Trastuzumab)
ESR1 (ER)	Estrogen Receptor 1	Predictive/Prognostic	Endocrine therapy (e.g., Tamoxifen) eligibility
MKI67 (Ki-67)	Marker of Proliferation Ki-67	Prognostic	Cell proliferation rate, tumor grade, recurrence risk
PIK3CA	Phosphatidylinositol-4,5-bisphosphate 3-kinase	Predictive	Targeted therapy with Alpelisib in HR+/HER2− cases
PGR (PR)	Progesterone Receptor	Predictive/Prognostic	Endocrine therapy response, often linked to ER
TP53	Tumor Protein p53	Risk/Prognostic	Frequent mutation in TNBC; DNA repair, apoptosis regulator

**Table 5:** Other diagnostic biomarkers for breast cancer based on their source (serum, urine, or tissue).

Type	Biomarker	Description/Role
Serum-Based	Autoantibodies (e.g., p53, MUC1)	Detectable before clinical symptoms in some patients; investigated as biomarkers for early breast cancer detection [59]
	CA 15-3/CA 27-29	Serum tumour markers measuring circulating MUC1-related antigens. They may assist in monitoring treatment response and disease progression in metastatic breast cancer but are not recommended for breast cancer screening, diagnosis, staging, or routine surveillance after curative treatment [60,61].
	CEA (Carcinoembryonic Antigen)	Carcinoembryonic antigen (CEA) is a glycoprotein tumour-associated marker that may be elevated in some patients with breast cancer, particularly those with advanced or metastatic disease. It is primarily used as an adjunctive biomarker for monitoring disease progression and response to systemic therapy when interpreted alongside clinical findings and imaging [60,61].
	Circulating tumour DNA (ctDNA)	Enables detection of actionable genomic alterations, minimal residual disease, treatment response, and emerging resistance through liquid biopsy [44,62].
	HE4 (Human Epididymis Protein 4)	A secreted glycoprotein investigated as a potential serum biomarker in breast cancer. Although elevated HE4 levels have been reported in some patients and may provide diagnostic or prognostic information when combined with other biomarkers, HE4 is not currently recommended for routine breast cancer diagnosis or monitoring [63].
	HER2 Extracellular Domain (HER2-ECD)	A soluble circulating fragment of the HER2 receptor released into the bloodstream through extracellular shedding. Elevated HER2-ECD levels have been investigated as a potential biomarker for monitoring response to HER2-targeted therapies and disease progression, although routine clinical application remains limited [60,64].
	Circulating miRNAs (e.g., miR-21, miR-155, miR-29a)	Small non-coding RNAs detectable in blood that regulate gene expression and tumour-related pathways. Circulating miRNAs are promising minimally invasive biomarkers for breast cancer detection, prognosis, treatment response, and molecular subtype classification, including triple-negative breast cancer; however, clinical translation remains limited by assay standardisation and biological variability [65].
	SEMA4C (Semaphorin 4C)	A member of the semaphorin family involved in tumour–microenvironment interactions, cell migration, angiogenesis, and metastatic progression. SEMA4C has shown potential as an emerging biomarker associated with breast cancer progression; however, its diagnostic utility and clinical application require further validation in larger patient cohorts [66].
Tissue-Based	BRCA1/BRCA2	Germline or somatic alterations in BRCA1/2 impair homologous recombination DNA repair and contribute to hereditary breast cancer risk. Identification of pathogenic BRCA mutations supports risk assessment and guides the use of PARP inhibitors, particularly in HER2-negative breast cancer [61,67].
	Cyclin D1 (CCND1)	A key regulator of the G1/S cell-cycle transition that is frequently amplified or overexpressed in hormone receptor-positive/luminal breast cancers. Altered CCND1 signalling has been associated with tumour proliferation, endocrine resistance, and prognosis [61].
	EpCAM (Epithelial Cell Adhesion Molecule)	Cell adhesion molecule expressed in epithelial cancers, including breast cancer. Used as a marker for circulating tumour cell detection in liquid biopsy approaches and investigated as a potential therapeutic target; however, its reliability may be affected by tumour heterogeneity and epithelial–mesenchymal transition [68].
	ER (Estrogen Receptor)	A key predictive and prognostic biomarker expressed in approximately 70–80% of breast cancers. ER positivity identifies patients who are likely to benefit from endocrine therapy and is central to breast cancer classification and treatment decision-making [60,61].
	HER2 (Human Epidermal Growth Factor Receptor 2)	A predictive biomarker for HER2-targeted therapies. HER2 overexpression or amplification identifies patients eligible for anti-HER2 treatment. Status is routinely assessed using immunohistochemistry (IHC) and confirmed by *in situ* hybridisation (ISH/FISH) when required [60,69].
	Ki-67	A nuclear proliferation marker assessed by immunohistochemistry. Ki-67 provides prognostic information and may aid in distinguishing luminal A-like and luminal B-like breast cancer subtypes; however, clinical interpretation is limited by variability in scoring and assay standardisation [70,71].
	Mammaglobin-A	Breast-specific secretory protein used as an immunohistochemical marker to support identification of breast carcinoma, including metastatic breast cancer; however, expression may occur in some non-breast malignancies and requires interpretation alongside other markers [72].
	MammaPrint (70-gene signature)	A multigene expression assay that categorises early-stage breast cancers according to genomic recurrence risk. It assists adjuvant chemotherapy decision-making by identifying patients with a low likelihood of distant recurrence who may derive limited benefit from chemotherapy [60,61].
	Oncotype DX (21-gene recurrence score)	A multigene expression assay used in early-stage ER-positive, HER2-negative breast cancer to estimate recurrence risk and predict the likely benefit of adjuvant chemotherapy. It supports personalised treatment decisions by identifying patients who may safely avoid chemotherapy while maintaining favourable outcomes [54,60].
	PR (Progesterone Receptor)	A predictive and prognostic biomarker reflecting functional hormone receptor signalling. PR expression supports classification of hormone receptor-positive breast cancer and is associated with endocrine therapy responsiveness and improved prognosis [60,61,71].
Urine-Based	Cyclic GMP (cGMP)	An investigational urinary metabolite explored as a potential indicator of tumour-associated metabolic alterations. Although metabolomic studies suggest potential value for early detection and disease monitoring, clinical validation and standardisation remain necessary before routine application [73].
	MAST4 (Microtubule-associated serine/threonine kinase family member 4)	An emerging urinary protein biomarker identified through proteomic approaches and investigated for its potential role in early breast cancer detection. However, evidence remains preliminary, and further validation in larger clinical cohorts is required before routine clinical application [73].
	Urinary metabolites (e.g., N-(2-furoyl)glycine)	Emerging non-invasive biomarkers identified through metabolomic profiling of blood and urine samples. These metabolic signatures have shown potential for early breast cancer detection, molecular classification, and prediction of therapeutic response; however, further external validation and clinical standardisation are required before routine application [74,75].
	Urinary miRNAs (e.g., miR-424, miR-423)	Emerging non-invasive biomarkers detectable in body fluids and investigated for early breast cancer detection and molecular profiling. Preliminary studies demonstrate diagnostic potential; however, variability in sample processing, assay methods, and the need for large-scale clinical validation currently limit routine clinical implementation [76,77,78].

**Table 6:** Overview of conventional, targeted, immune-based, and emerging therapeutic strategies in breast cancer management.

Treatment Category	Strategy/Agents	Mechanism of Action	Clinical Implication	Key Limitations
Conventional Treatment				
1. Surgery	Lumpectomy (breast-conserving surgery)	Physical removal of the primary tumour while preserving breast tissue; commonly followed by radiotherapy to reduce local recurrence [61].	Preferred surgical approach for many patients with early-stage breast cancer when tumour size, margins, and radiotherapy availability allow breast conservation [61,71].	Requires access to imaging, pathology, surgical expertise, and radiotherapy services. Limited access in LMICs contributes to greater reliance on mastectomy; risks include re-excision, recurrence, and cosmetic concerns [140,141]
	Mastectomy	Complete removal of breast tissue with assessment of regional lymph nodes when indicated [61].	Appropriate for extensive tumours, multifocal disease, recurrence, genetic risk reduction, or when breast conservation is unsuitable [71].	Surgical morbidity, psychological impact, need for reconstruction, and limited reconstructive services in resource-constrained settings [141].
2. Radiotherapy	External beam radiotherapy (EBRT), intensity-modulated radiotherapy (IMRT), hypofractionated radiotherapy	Causes tumour cell death through radiation-induced DNA damage and apoptosis [61].	Standard adjuvant therapy following breast-conserving surgery and selected mastectomy cases; reduces local recurrence and improves survival outcomes [61].	Global disparities in access due to inadequate radiotherapy infrastructure, workforce shortages, treatment delays, and acute/late toxicities [96,142].
3. Chemotherapy	Anthracyclines, taxanes, platinum agents	Induces DNA damage, inhibits replication, disrupts microtubule function, and promotes apoptosis [71].	Used in neoadjuvant, adjuvant, and metastatic settings; improves survival and increases eligibility for breast-conserving surgery through tumour downstaging [86,143].	Toxicity including cardiotoxicity, neuropathy, myelosuppression, treatment intolerance, and acquired resistance limit effectiveness [71].
4. Hormonal Therapy	Tamoxifen, Aromatase inhibitors, Fulvestrant	Blocks oestrogen receptor signalling or inhibits peripheral oestrogen synthesis, reducing hormone-dependent tumour proliferation [61].	Standard treatment for ER-positive breast cancer; improves recurrence-free and overall survival in early and metastatic disease [61,71].	Endocrine resistance, menopausal symptoms, osteoporosis, thromboembolic risk, and adherence challenges affect long-term effectiveness [71].
Targeted and Immune-Based Treatment				
1. Targeted Therapy	HER2 inhibitors (Trastuzumab, pertuzumab, T-DM1), CDK4/6 inhibitors, PI3K/AKT/mTOR inhibitors	Selectively inhibit molecular pathways responsible for tumour proliferation and survival [71].	Enables subtype-specific therapy; HER2-targeted therapies significantly improve outcomes in HER2-positive disease, while CDK4/6 inhibitors improve progression-free survival in HR-positive/HER2-negative disease [61,117].	Biomarker dependency, high treatment cost, acquired resistance, cardiotoxicity, neutropenia, and metabolic adverse effects remain challenges [71].
2. Immunotherapy	Immune checkpoint inhibitors (Pembrolizumab, Atezolizumab)	Blocks PD-1/PD-L1 immune inhibitory signalling, restoring anti-tumour T-cell activity [30].	Provides benefit in selected TNBC patients, particularly PD-L1-positive tumours, usually in combination with chemotherapy [30].	Limited response rates, immune-related adverse events, lack of reliable predictive biomarkers, and accessibility issues limit widespread use [112].
3. Antibody–Drug Conjugates (ADCs)	Trastuzumab Deruxtecan (T-DXd), Trastuzumab Emtansine, Sacituzumab Govitecan	Delivers cytotoxic payloads selectively to tumour cells through antigen-specific antibodies [117].	Improves outcomes in HER2-positive, HER2-low, and metastatic TNBC populations [117,118].	Drug resistance, interstitial lung disease risk with some ADCs, cost, and complex manufacturing requirements [144].
Emerging and Precision Approaches				
1. PARP Inhibitors	Olaparib, Talazoparib	Exploits synthetic lethality by inhibiting DNA repair in BRCA-mutated or homologous recombination-deficient tumours [67,119].	Improves outcomes in germline BRCA-mutated HER2-negative early and metastatic breast cancer [67,119].	Resistance through restoration of homologous recombination, haematological toxicity, cost, and requirement for genetic testing [145].
2. Precision Medicine Approaches	Oncotype DX, MammaPrint, Prosigna, EndoPredict, NGS-guided therapy	Uses genomic profiling to predict recurrence risk and identify actionable molecular alterations [124].	Supports personalised treatment selection, chemotherapy de-escalation, and molecularly guided therapy [61].	High cost, limited molecular testing infrastructure, data interpretation complexity, and unequal access globally [79].
3. Epigenetic Therapy	DNA methyltransferase inhibitors and HDAC inhibitors	Reverses abnormal epigenetic modifications and restores tumour suppressor gene expression [146].	Potential application in resistant breast cancer and aggressive molecular subtypes [146].	Mostly early-phase clinical development, uncertain efficacy, toxicity concerns, and lack of validated predictive biomarkers [147].
4. Tumour Microenvironment (TME)-Targeted Therapy	CAF inhibitors, anti-angiogenic agents, macrophage-targeting therapies	Modulates stromal, immune, and vascular components supporting tumour progression [133].	May overcome resistance and improve response to immunotherapy and targeted therapies [134].	Biological complexity of the TME, tumour heterogeneity, and limited clinical validation remain barriers [135].
5. Personalised Vaccines and mRNA Platforms	Neoantigen vaccines, personalised mRNA vaccines	Activates tumour-specific immune responses against patient-specific cancer antigens [148].	Potential for durable immune control and combination with immunotherapy or conventional treatment [135].	Early clinical development, manufacturing complexity, high cost, immune escape, and limited breast cancer-specific evidence [148].
6. Nanotechnology and Advanced Drug Delivery Systems	Liposomes, polymer nanoparticles, exosomes	Improves drug delivery through tumour targeting, enhanced pharmacokinetics, and reduced systemic toxicity [149].	Potential application for targeted delivery of chemotherapy, nucleic acids, and immune-modulating agents [150].	Manufacturing scalability, reproducibility, pharmacokinetic uncertainty, long-term safety concerns, and regulatory barriers [149].

**Table 7:** Common Mechanisms of Treatment Resistance in Breast Cancer.

Resistance Category	Key Molecular/Cellular Drivers	Affected Therapies	Impact on Treatment Outcome	Representative Recent Evidence (2020–2026)
Genetic Mutations	*ESR1* activating mutations; *PIK3CA*, *AKT1*, *ERBB2* mutations; BRCA1/2 reversion mutations	Endocrine therapy, HER2-targeted agents, PARP inhibitors	Constitutive signaling, bypass of drug targets, restoration of DNA repair	[188,201,202]
Epigenetic Alterations	DNA hypermethylation; histone modifications (HDAC activation); chromatin remodeling	Endocrine therapy, chemotherapy, immunotherapy	Silencing of tumor suppressors; induction of stem-like, drug-tolerant states	[203]
Adaptive Signaling Rewiring	PI3K/AKT/mTOR activation; MAPK pathway upregulation; FGFR, IGF-1R cross-talk	Endocrine therapy, HER2-targeted therapy	Bypass of inhibited receptors; sustained proliferation and survival	[188,202]
Cell Cycle Dysregulation	Cyclin D overexpression; CDK activation; RB pathway alterations	Endocrine therapy, CDK4/6 inhibitors	Continued cell cycle progression despite therapy	[184,204]
DNA Damage Repair Adaptation	Restoration of homologous recombination; replication fork stabilization	PARP inhibitors, chemotherapy	Loss of synthetic lethality; reduced DNA damage–induced apoptosis	[189]
Tumor Microenvironment Protection	Cancer-associated fibroblasts; tumor-associated macrophages; hypoxia	Chemotherapy, targeted therapy, immunotherapy	Drug shielding, immune suppression, reduced drug penetration	[205,206,207]
Immune Evasion	PD-L1 upregulation; regulatory T cells; myeloid-derived suppressor cells	Immune checkpoint inhibitors	Primary and acquired immunotherapy resistance	[205,206,207]
Pharmacokinetic Resistance	CYP450 polymorphisms (e.g., CYP2D6); increased drug efflux (ABC transporters)	Tamoxifen, chemotherapy	Reduced active drug levels and therapeutic exposure	[181,182,183]

**Table 8:** Some examples of clinical studies on breast cancer gene therapy (data obtained from www.clinicaltrials.gov accessed on 23 February 2026).

Description	Condition	Trial Phase	Study Record Updates	National Clinical Trial (NCT) Number	Location of Study
Adenovirus-mediated human interleukin12	Breast cancer	Phase One	Completed	NCT00849459	Icahn Medical Center at Mount Sinai New York, New York, United States
Ad5CMV-p53 gene + chemotherapy	Breast cancer	Phase One	Completed	NCT00004038	Fox Chase Cancer Center Philadelphia, Pennsylvania, United States
Ad5CMV-p53 gene+ drug: docetaxel, doxorubicin hydrochloride	Breast cancer	Phase Two	Completed	NCT00044993	University of Texas—MD Anderson Cancer Center
AdV-tk + valacyclovir	Breast, lung and ovarian cancer, Mesothelioma, Malignant Pleural Effusion	Phase One	Active, not recruiting	NCT01997190	University of Pennsylvania, United States
CD34+ Derived Dendritic Cells Transduced with an Adenovirus Vector Expressing Inactivated HER-2/Neu	Breast cancer	Phase One	Completed	NCT00197522	Hamilton Health Sciences Hamilton, Ontario, Canada
Retroviral Transduction of the MDR1 gene Into Peripheral Blood Progenitor Cells (PBPCs)	Breast cancer, Neoplasm metastasis	Phase Two	Completed	NCT00001493	National Cancer Institute (NCI) Bethesda, Maryland, United States

## Data Availability

No new data were created or analyzed in this study. Data sharing is not applicable to this article.
